# A distinct strain of *Arsenophonus* symbiont decreases insecticide resistance in its insect host

**DOI:** 10.1371/journal.pgen.1007725

**Published:** 2018-10-17

**Authors:** Rui Pang, Meng Chen, Lei Yue, Ke Xing, Tengchao Li, Kui Kang, Zhikun Liang, Longyu Yuan, Wenqing Zhang

**Affiliations:** 1 State Key Laboratory of Biocontrol, School of Life Sciences, Sun Yat-sen University, Guangzhou, China; 2 State Key Laboratory of Applied Microbiology Southern China, Guangdong Provincial Key Laboratory of Microbial Culture Collection and Application, Guangdong Open Laboratory of Applied Microbiology, Guangdong Institute of Microbiology, Guangzhou, China; University of Michigan, UNITED STATES

## Abstract

Symbiotic bacteria are important drivers of phenotypic diversity in insects. One of the widespread symbionts to have emerged belongs to the genus *Arsenophonus*, however, its biological functions in most host insects remain entirely unknown. Here we report two distinct *Arsenophonus* strains in the brown planthopper (BPH), *Nilaparvata lugens*, a major pest insect in Asian countries that causes significant economic damage through rice crop destruction. Genomic resequencing data suggested that one *Arsenophonus* strain (S-type) negatively affected the insecticide resistance of the host. Indeed, replacement of the resident *Arsenophonus* with the S-type *Arsenophonus* significantly decreased host insecticide resistance. Transcriptome and metabolome analysis revealed down-regulation of xenobiotic metabolism and increased amino acid accumulation in the S-type *Arsenophonus* infected host. This study demonstrates how a symbiont-mediated phenotypic change can occur. The results of this study will aid in developing strategies that work through imposing an ecological disadvantage on insect pests, which will be of great value for pest control in agricultural industry.

## Author summary

Symbiotic bacteria are key evolutionary drivers, providing innovations that allow their eukaryotic hosts to evolve new phenotypes. Beneficial traits provided by the acquisition of a specific symbiont have been reported in many kinds of insect host, however the potential of the symbiont infection to confer an ecological disadvantage to the insect host has received little attention. Here we describe a novel kind of symbiotic relationship between an herbivorous insect pest, *Nilaparvata lugens*, and its facultative symbiont *Arsenophonus*. We identified two distinct *Arsenophonus* strains in this insect. One strain had been previously reported (N-type), while the other, rarely observed strain presented a relatively sensitizing effect through reduction of insecticide resistance (S-type). Infection with the S-type *Arsenophonus* caused down-regulation of xenobiotic metabolism, which may explain the sensitized effect, while in turn serving to increase amino acid accumulation in the host as a putative trade-off.

## Introduction

Bacterial symbionts play important roles in the diversification of eukaryotes [1]. Many organisms rely on symbionts for physiological functions and some symbiotic relationships can even drive the development of important phenotypes [2]. Insects in particular comprise the most diverse class of animals that harbor various bacterial symbionts and have established a wide diversity of symbiotic systems.

The relationship between the insect host and the symbiont can be defined as obligatory (essential) or facultative (non-essential). Bacterial symbionts can develop obligatory relationships with their hosts such that they are required for the survival of the host. For example, some symbionts provide essential nutrients lacking in the diets of the insects [3]. Most obligate symbionts play important roles in the reproduction of the host [4]. These symbionts are stably maintained by vertical transmission from mother to progeny in the host species, exhibiting co-evolutionary patterns with host lineages [5].

In contrast to obligate symbionts, facultative symbionts are not essential for host survival, but rather fulfill various functions for the host [4]. These types of symbionts occasionally undergo horizontal transmission within and between species [6]. Acquisition of horizontally transmitted symbionts allows hosts to diversify and acquire new traits. For example, some facultative symbiont species can determine the plant specialization of their herbivore hosts [7]. Infection of a particularly facultative symbiont in the pea aphid can provide resistance to parasitic wasps [8]. Likewise, some fenitrothion-degrading *Burkholderia* strains aid the stinkbug by conferring resistance to fenitrothion [9], while facultative *Hamiltonella defensa* can help the whitefly to overcome the induced plant defense [10]. Finally, replacement of a heat-sensitive *Buchnera* type with a heat-tolerant *Buchnera* type contributes to pea aphid matriline heat tolerance [11]. Notably, these acquired phenotypes may transfer to the progeny of infected hosts by vertical transmission, leading to new adaptive traits.

However, it is important to note that facultative symbionts do not always prove beneficial to the host, and can sometimes be deleterious. The genus *Arsenophonus* is an emerging clade of intracellular symbionts that demonstrate a vast host distribution. As a result, *Arsenophonus* is a typical model used to characterize the diverse roles of symbiotic associations [12]. *Arsenophonus* was first described for its ability to confer male-killing effect in the parasitoid wasp *Nasonia vitripennis* [13,14]. This deleterious effect was considered to be a strategy that enabled the spread of symbionts and their maintenance in host lineages through manipulation of host reproduction. However, such a phenomenon is rarely observed in other insect hosts of *Arsenophonus*. In *Bemisia tabaci* and *Aphis craccivora*, *Arsenophonus* infection was found to affect host plant specialization [15,16]. Other influences such as induced protection in the psyllid [17] and nutritional supplementation of the human body louse [18] have been reported, but not as general cases. It seems that different insect species may carry various *Arsenophonus* strains that confer distinct biological functions, and in some cases, a single insect species can contain multiple *Arsenophonus* strains [19]. Indeed, polygenetic analysis has shown a high diversity of this genus, indicating its potential evolutionary importance in insect hosts, but the influence of most *Arsenophonus* strains on host phenotype is entirely unknown [20]. Fortunately, the increasing availability of *Arsenophonus* genome sequences provides the opportunity to explore the ecological and evolutionary associations of this symbiont with different host lineages.

Here we studied the symbiosis between the brown planthopper *Nilaparvata lugens*, an herbivorous insect pest causing serious damages to rice crop in Asia, and its facultative symbiont *Arsenophonus*. The available genome sequences of *N*. *lugens* and its symbiont *Arsenophonus* make it an ideal system to explore the symbiont-host genetics [21]. Here we found two distinct *Arsenophonus* strains associated with different effects on host insecticide resistance. One strain is common in *N*. *lugens* [22] and does not affect insecticide resistance, while the other novel strain is rarely observed in most areas except for the Philippines and is associated with decreased resistance to insecticide. Replacement of the resident *Arsenophonus* with this novel *Arsenophonus* strain decreased host resistance to imidacloprid, an insecticide widely used for the control of *N*. *lugens*. Transcriptome and metabolome analysis revealed that the down-regulation of xenobiotic metabolism likely accounts for the increased susceptibility to insecticides. Importantly, our study demonstrates a symbiont-mediated phenotypic change that confers an ecological disadvantage to the host. This finding holds great potential value for the development of pest control strategies in the agricultural industry.

## Results and discussion

### Discovery of a distinct *Arsenophonus* strain

**Fig 1:**
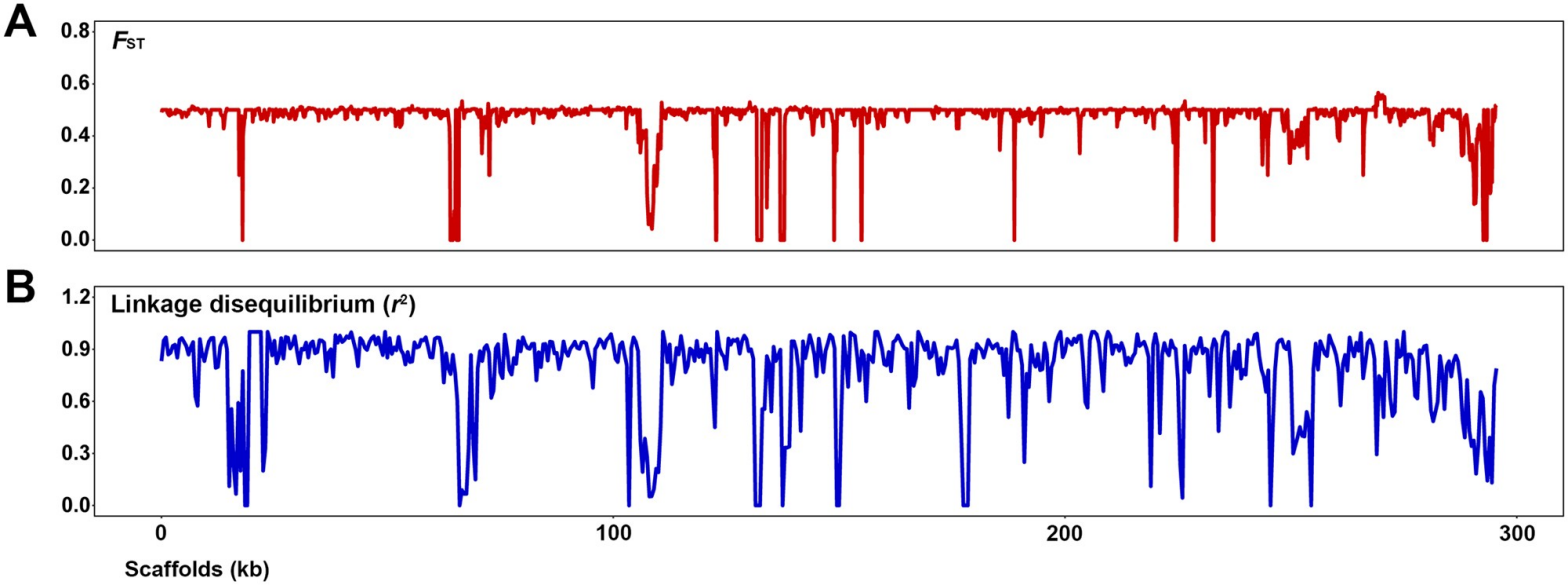
Divergence in the genomic landscape of *Arsenophonus* from resistant and susceptible *N*. *lugens*. **(A)** Pairwise genetic differentiation (*F*_ST_) across *Arsenophonus* genomes from resistant and susceptible *N*. *lugens* in 5-kb windows with 1-kb sliding steps. **(B)** The genome-wide linkage disequilibrium (LD) of variations in susceptible samples across the *Arsenophonus* genome in 5-kb sliding windows.

We previously searched for mutations associated with insecticide resistance by comparing whole-genome sequences of insecticide-resistant and—susceptible individuals from a field *N*. *lugens* population collected from Guangxi, China in 2011 (see details in Materials and methods). Genomic resequencing analysis of insects resistant or susceptible to imidacloprid, a typical neonicotinoid insecticide, identified multiple fragments of the *Arsenophonus* endosymbiont genome. We then compared the genetic diversity between *Arsenophonus* from resistant and susceptible *N*. *lugens* by estimating the pairwise *F*_ST_ value using a sliding window approach (5-kb windows sliding in 1-kb steps) according to the allelic frequencies of genome-wide single nucleotide polymorphisms (SNPs) (Fig 1A). Unexpectedly, the average pairwise *F*_ST_ value was 0.4712 across the whole genome of *Arsenophonus*, which indicated the existence of an extremely high genetic diversity [23]. In addition, the distribution of *F*_ST_ values was disproportionate (S1 Fig). The *F*_ST_ value of most regions was about 0.50, only a few regions presented *F*_ST_ values within the range of 0.01 to 0.30 (2.03%), and 1.60 percent of the regions showed no difference between the two groups. Thus, with the exception of only a few regions, significant differences were present across the entire genome of *Arsenophonus* from resistant and susceptible insects. Notably, only a few SNPs were found in the *Arsenophonus* genome from resistant *N*. *lugens* compared to the reference *Arsenophonus* draft genome (GCF_000757905.1) (Table 1). The high degree of sequence similarity (> 99.9%) suggested that resistant insects exactly carried the same *Arsenophonus* strain as the reference one. In contrast, a large amount of SNPs with an average allelic frequency of 0.47 were identified in the *Arsenophonus* genome from susceptible *N*. *lugens*, reflecting the existence of more than one *Arsenophonus* strain in susceptible insects.

**Table 1:** Statistics of single nucleotide polymorphisms (SNPs) across *Arsenophonus* genome from resequencing data.

Sample	IR	IS
SNP number	1,166	19,249
Average depth of SNPs	95.91	40.35
SNP rate per 100 bp	0.04	0.65
Exon SNPs	1,073	16,176
Missense SNPs	384	6,404
Start codon lost	0	7
Stop codon lost	2	16
Premature termination codon	5	89

IR: Imidacloprid-resistant samples; IS: Imidacloprid-susceptible samples.

To characterize the distribution of SNPs in the *Arsenophonus* genome from susceptible *N*. *lugens*, we calculated the genome-wide linkage disequilibrium (LD) of these SNP variations using VCFTools software. The results revealed a strong LD among all SNPs in each scaffold (R^2^>0.33), and the SNPs in most scaffolds were nearly in complete LD (Fig 1B). In addition, the distribution of LD value was highly correlated with the pattern of *F*_ST_ value. This indicated that all *Arsenophonus* polymorphisms presented in the susceptible *N*. *lugens* exactly belonged to an *Arsenophonus* strain distinct from the previously reported one. We validated this inference by performing PCR amplification and sequencing of randomly selected SNPs. For any defined individual, if one site was detected to be the new-found allele, then all other sites must be new-found alleles (S2 Fig).

So far, we found two *Arsenophonus* strains in susceptible *N*. *lugens*. For convenience, we named the previously reported strain as N-type *Arsenophonus*, and the newly discovered strain as S-type *Arsenophonus*. The unique presence of S-type *Arsenophonus* in susceptible individuals suggested that this strain may be related to the insecticide susceptibility of its host insects. To support this inference, we compared the types of *Arsenophonus* strains from *N*. *lugens* individuals that were resistant or susceptible to another category of widely used insecticide, buprofezin. We observed that only the buprofezin-susceptible samples carried S-type *Arsenophonus*, while N-type *Arsenophonus* was presented in both resistant and susceptible insects (S1 Table, S3 Fig). These results suggest that the S-type *Arsenophonus* may affect host susceptibility to more than one chemical insecticides.

Previous work has suggested different *Arsenophonus*-host relationships ranging from parasitism to mutualism [20], including male-killing [14] and obligatory nutrient supplement [18]. In *N*. *lugens*, whereas no significant effect on reproductive manipulation from *Arsenophonus* infection was found [24], genomic analysis indicated a potential role of N-type *Arsenophonus* in synthesizing B vitamins for the host [22]. We found that B vitamin synthesis was not affected by the new-found alleles, suggesting that the obligatory function for the host is supported by both strains. However, the SNPs in the S-type strain led to a premature termination codon (PTC) in 89 genes that were enriched in functional categories related to protein modification and response to chemical stimulus (Table 1, S4 Fig), suggesting that S-type *Arsenophonus* may lose some regulatory functions under the chemical stimulus. This is also in agreement with our inference that the S-type strain may confer a disadvantage to host insecticide resistance.

### Geographical distribution of S-type *Arsenophonus* in *N*. *lugens* populations

**Fig 2:**
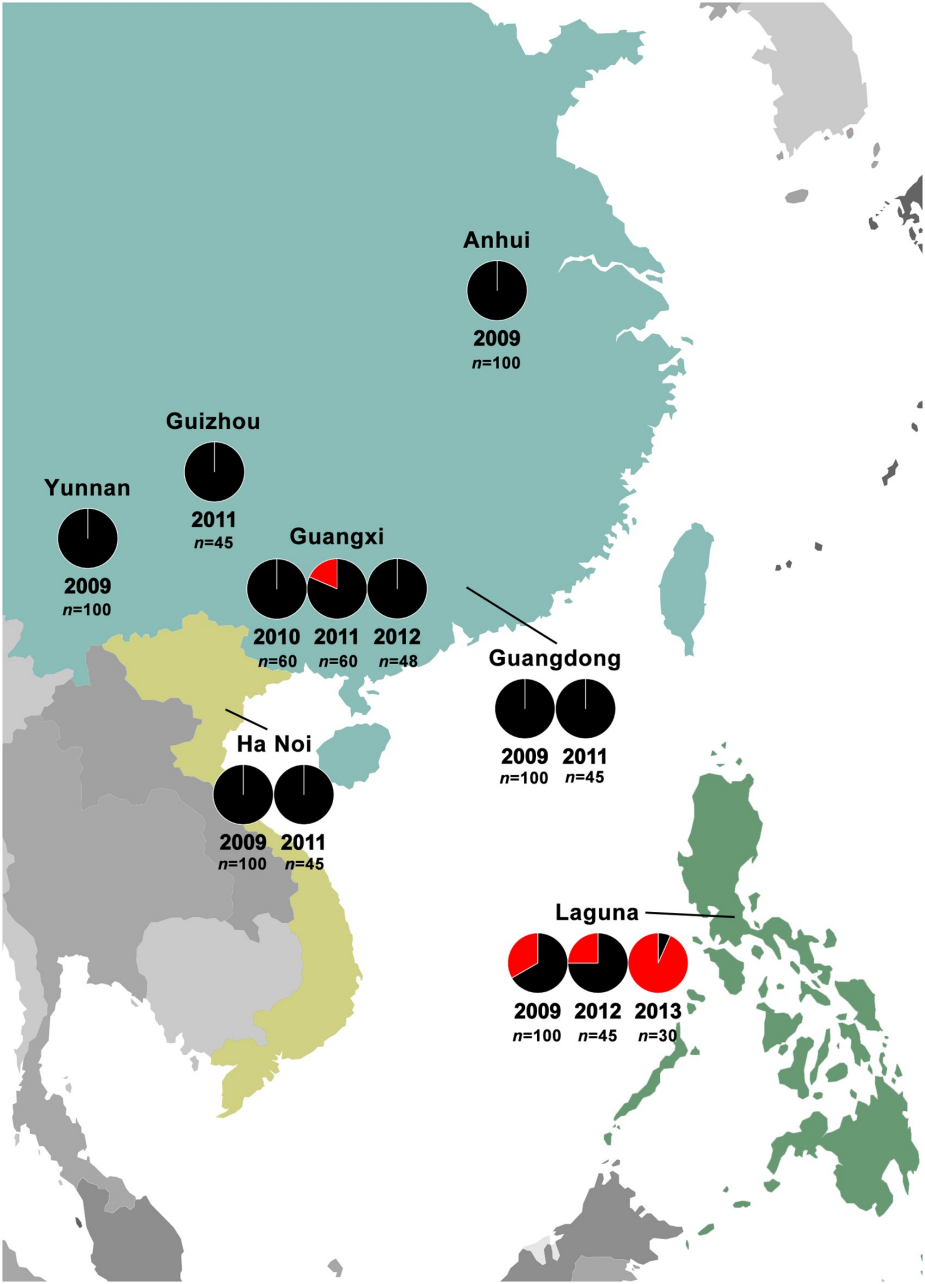
Infection frequencies with S-type (red) and N-type (black) *Arsenophonus* in *N*. *lugens* populations from different areas. The years of collection and numbers of tested individuals are also shown.

We traced the distribution of S-type *Arsenophonus* by genotyping of *Arsenophonus* in *N*. *lugens* populations collected from multiple areas in different years (Fig 2, see details in S2 Table). In China, with the exception of the Guangxi population collected in 2011, no *N*. *lugens* population was found to carry S-type *Arsenophonus*. Similarly, no S-type *Arsenophonus* was found in *N*. *lugens* populations from Vietnam. To our surprise, S-type *Arsenophonus* was detected in all populations from the Philippines collected in different years. Why the S-type *Arsenophonus* was present in Guangxi population, 2011 remains a mystery, however it appears clear that S-type *Arsenophonus* was widespread among *N*. *lugens* populations in the Philippines. Current studies on the migration of *N*. *lugens* indicate that *N*. *lugens* present in east Asia originated from the Indochina peninsula, and thus are geographically isolated from populations in the Philippines [25,26]. This may partly explain the differences in the distribution of S-type *Arsenophonus* between the Philippines and other areas. Considering that China and Vietnam are two of the largest consumers of insecticide for rice crop protection (statistics from Phillips McDougall), it sounds reasonable that S-type *Arsenophonus* did not spread in these areas.

### Change in the proportion of S-type *Arsenophonus* in response to insecticide selection

**Fig 3:**
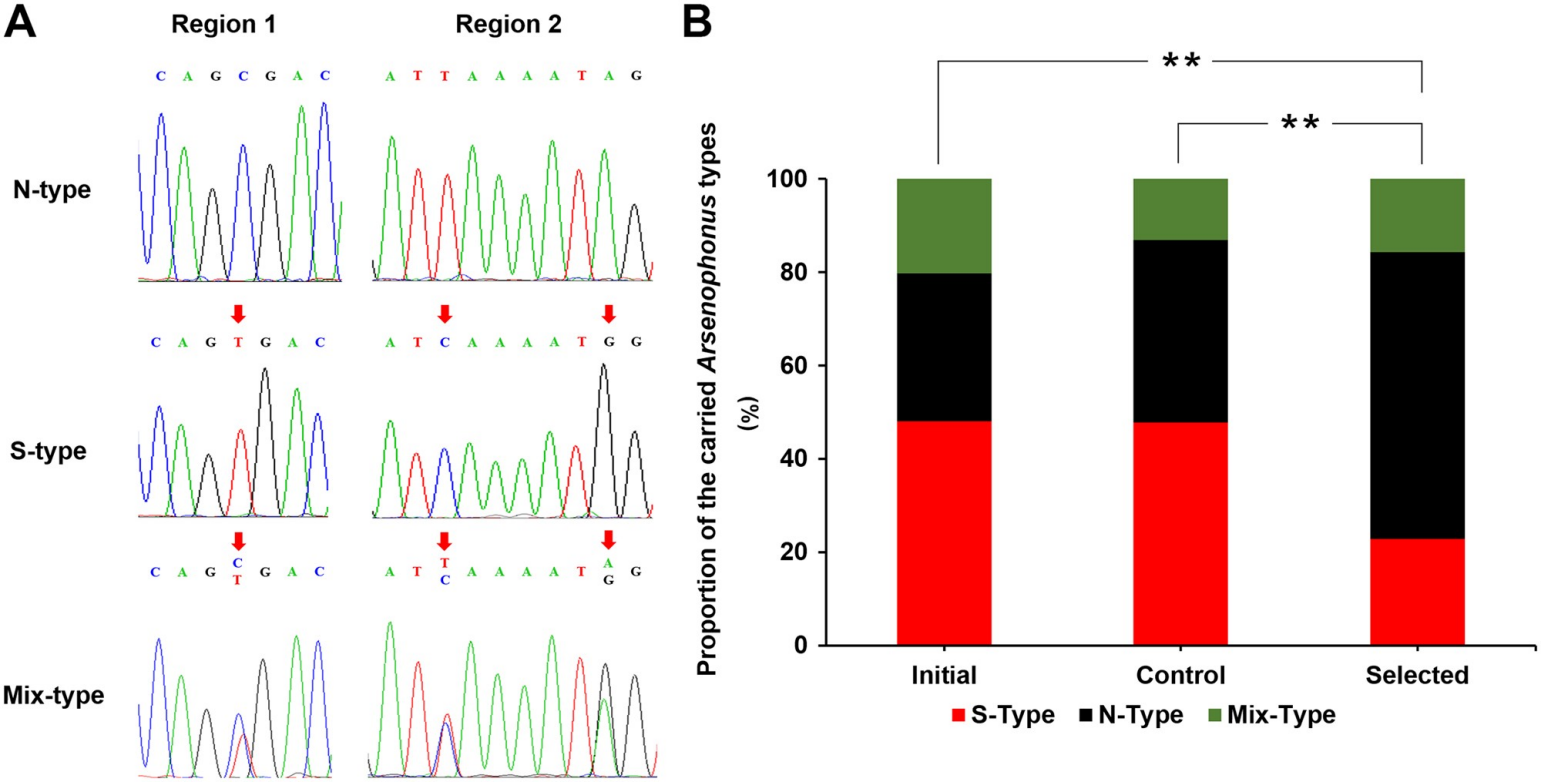
Insecticide selection affects the proportion of *Arsenophonus* types that *N*.*lugens* carried. **(A)** Sequencing chromatograph showing the characterization of N-type, S-type, and Mix-type *Arsenophonus*. N-type chromatogram indicates that the insect individual dominantly carries N-type *Arsenophonus*, S-type chromatogram reveals that the insect individual dominantly carries S-type *Arsenophonus*, while Mix-type chromatogram reflected that the insect individual is co-infected with both *Arsenophonus* strains evenly. **(B)** Change in the proportion of carried *Arsenophonus* strains in the Philippines *N*. *lugens* population under the selection of imidacloprid. Significance analysis was performed based on the frequencies of S-type *Arsenophonus*. Initial sample: n = 79; Control sample: n = 69; Selected sample: n = 70. **: *P* < 0.01 (*u*-test).

To begin to explore the effect of S-type *Arsenophonus* on a population, we genotyped a field *N*. *lugens* population collected from Laguna, Philippines in 2015. One or the other *Arsenophonus* strains could be detected in most individuals of this population (S3 Table). Moreover, a portion of individuals carried both types of *Arsenophonus* (Fig 3A), indicating the co-infection of N-type and S-type *Arsenophonus* in a single insect (Mix-type). Notably, deep-sequencing (Hiseq4000, PE150, 100 × coverages) of the Philippines-collected *N*. *lugens* that was considered to carry only S-type *Arsenophonus* (as determined by Sanger sequencing) could detect N-type bacterium at a very small scale (~ 8.59%, S5 Fig), revealing a detection limitation of Sanger sequence chromatographs. Horizontal transmission of different *Arsenophonus* strains occurs repeatedly among plants hosting aphids [27], and insect–plant interactions are also a potential route for horizontal transmission [28]. However, the coinfection of distinct *Arsenophonus* strains in tested *N*. *lugens* population seemed to be the norm rather than a recently horizontal transmission event, with some individuals possessed one or the other dominant strain. Given that the titer of N-type *Arsenophonus* was below detection level by chromatographic inspection in S-type insects that was susceptible to insecticide, resistance phenotype of these insects was likely determined by the dominant *Arsenophonus* strain. Therefore, we continued to use the Sanger sequence chromatographs to determine whether individual insects dominantly carried S-type or N-type strain (Fig 3A). For example, N-type chromatogram indicated that the titer of S-type *Arsenophonus* was inadequate to affect host’s phenotype, while Mix-type chromatogram reflected that the corresponding insect individual were completely co-infected with both *Arsenophonus* strains.

To test the sensitized effect of S-type *Arsenophonus*, we randomly selected 79 mated female adults from the Philippines population (2015) and performed *Arsenophonus* genotyping one week after spawning. The offspring of these females were pooled together and then randomly divided into two groups. One group was fed normally (control), while the other group was challenged with 10 mg/L imidacloprid according to its LD_50_ (selected). After continuous selection for 3 generations, the proportion of S-type *Arsenophonus* in selected insects (22.86%) was significantly lower than in the initial (48.10%) and control insects (47.83%), whereas no substantial change was observed between the control and the initial insects (Fig 3B). These results indicated that the S-type *Arsenophonus* is not conducive to the survival of *N*. *lugens* exposed to insecticide treatment.

### Effect of S-type *Arsenophonus* on host resistance to imidacloprid

**Fig 4:**
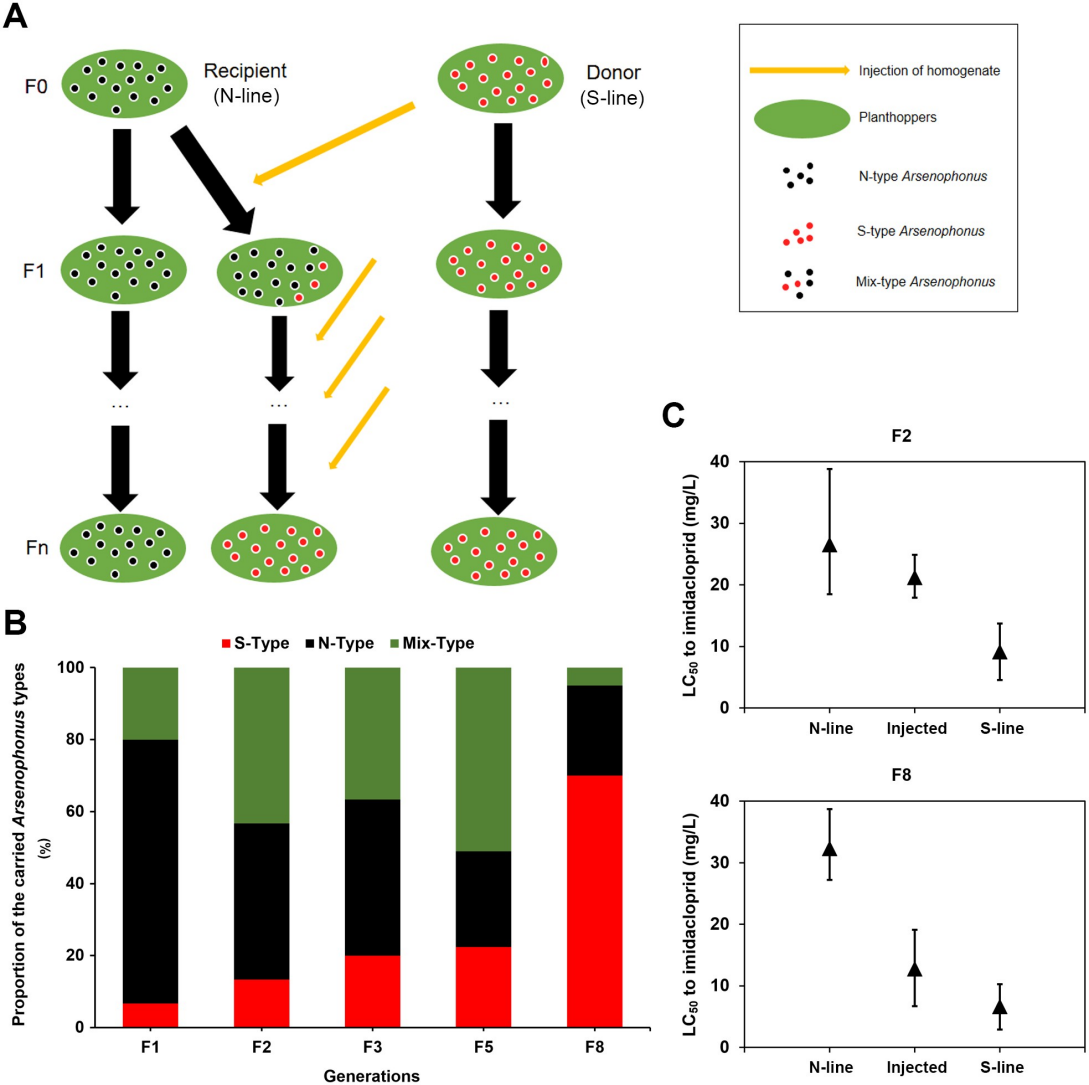
Replacement of the N-type *Arsenophonus* in *N*. *lugens* with S-type *Arsenophonus* impacts the host’s insecticide resistance. **(A)** Experimental approach for replacement of the native *Arsenophonus* within a *N*. *lugens* matriline. The recipient (N-line) contains N-type *Arsenophonus*, and the donor (S-line) contains S-type *Arsenophonus*. Microinjection is used to flood the hemocoel with donor *Arsenophonus*, and the injection is conducted on each subsequent generation until the generation of F8 progeny. **(B)** Changes in the proportion of carried *Arsenophonus* strains during the replacement process. **(C)** Insecticide resistance of *N*. *lugens* during the replacement process. The untreated N-line and S-line were used as controls. The data is shown as 50% lethal concentration (LC_50_), and bars represent the range of 95% confidence limit (CL).

Experimental techniques such as microinjection, antibiotic therapy and introgression have been applied to test the associations in different symbiont-insect systems [29,30]. However, introgression between *Arsenophonus*-infected and—uninfected insect lines is rarely reported. While the technique of antibiotic manipulation, which may not only remove the target bacteria but also affect the whole symbiont community composition [31], do not suit for the investigation of the relationship between S-type *Arsenophonus* and host’s phenotype. Thus, we tested the effect of replacing resident *Arsenophonus* with distinct *Arsenophonus* strain from a different host matriline, by using a protocol of homogenate injection on the basis of the approach described by Moran and Yun [11] (Fig 4A, see details in Materials and methods). Two *N*. *lugens* lines that dominantly carried either S-type or N-type *Arsenophonus* were screened by pooling offspring from multiple isofemale lines. The N-line *N*. *lugens* (dominantly carrying N-type strain) was selected as the recipient, and the S-line *N*. *lugens* (dominantly carrying S-type strain) was selected as the donor. The first injection resulted in 6.67% and 20.00% of individuals containing S-type and Mix-type *Arsenophonus*, respectively (Fig 4B). After injection of subsequent generations, the proportion of S-type *Arsenophonus* in the progeny of injected females had increased. By the injection of eighth generation, most of the N-type *Arsenophonus* in the progeny of the recipient had been replaced with the S-type and Mix-type *Arsenophonus* (> 75%).

As a result, the 50% lethal concentration (LC_50_) of imidacloprid for the injected line decreased significantly from F2 (21.187 mg/L) to F8 (12.785 mg/L) (Fig 4C). In the second generation after injection, the resistance level of the injected line was still close to that of the N-line (26.550 mg/L) and significantly higher than that of S-line (9.147 mg/L). However, after replacement of the majority of N-type by injection of S-type strain, the injected line demonstrated a low level of imidacloprid resistance, similar to the S-line (6.622 mg/L) and with a LC_50_ much lower than that of N-line (32.318 mg/L). These results showed that replacement of *Arsenophonus* with S-type strain indeed decreased insecticide resistance in the N-line *N*. *lugens*.

**Fig 5:**
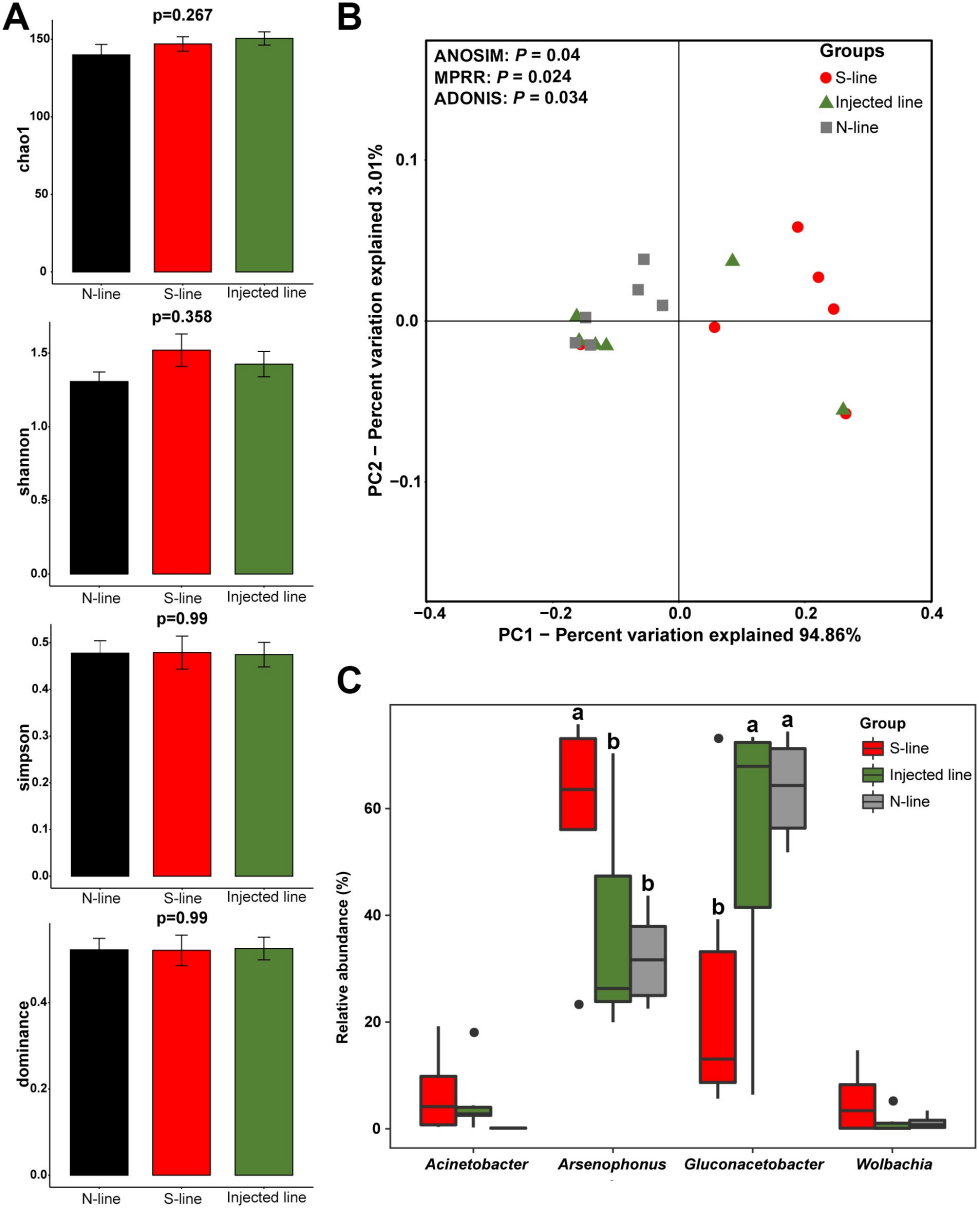
Comparison on the bacterial communities among N-line, S-line and injected line *N*. *lugens*. No significant difference is observed in α diversity (measured by Chao1, Shannon, Simpson, and dominance) of bacterial communities among three insect lines (A), while β diversity of bacterial communities presents significant difference between different insect lines according to the PCoA and Anosim MRPP, and Adonis test (B). (C) The differences in relative abundance of core bacterial genera among three insect lines are summarized. The values sharing the same letter are not significantly different at *P* < 0.05 (Tukey’s range test).

One concern is that if the phenotypic change of host insect was attributed to the replacement of *Arsenophonus* strain rather than the bacteria community. To include the influences of other co-infecting symbiotic bacteria, we analyzed the diversity of bacteria communities in insects from N-line, S-line, and injected line, using the Illumina HiSeq platform targeting the V3–V4 regions of 16S rDNA. The bacterial diversity and relative abundance of all samples in the different taxonomy are presented in S6 Fig. Alpha diversity was applied to compare the species diversity among different sample lines. According to the α diversity parameters of each sample, including Chao1, Shannon, Simpson, and dominance, no significant difference was observed in the bacterial community composition of samples from N-line, S-line, and injected line (Fig 5A). Further analysis revealed that S-line and N-line differed in the β diversity of bacterial composition, which was measured by Principal Coordinate Analysis (PCoA) and tested with Anosim (analysis of similarities, *P* = 0.04), MRPP (Multi Response Permutation Procedures, *P* = 0.024) and Adonis (permutational MANOVA, *P* = 0.034) methods, however, the injected line presented no significant differences to N-line in bacteria communities (Fig 5B). Of all detected genera, *Arsenophonus* and three other genera, *Gluconacetobacter*, *Acinetobacter* and *Wolbachia*, occupied more than 94% of all bacteria in every sample (S6 Fig, S4 Table), which could be considered as core bacterial genera in tested lines. The core bacterial symbionts were also in accordance with the previous finding in *N*. *lugens* [32]. We then compared the relative abundance of these genera among three insect lines. There was no significant difference in the relative abundance of *Acinetobacter* and *Wolbachia* among three lines (Fig 5C), while *Arsenophonus* was significantly decreased from S-line to N-line and injected line, and *Gluconacetobacter* was opposite. However, the relative abundance of core bacterial genera did not show any significant difference between injected line and N-line. These findings reflected that the homogenate injection did not fundamentally affect bacterial diversity and relative abundance in the recipient insects, at least till F8 generation.

Although the difference was not significant in statistics, the relative abundance of the two major genera (*Arsenophonus* and *Gluconacetobacter*) in the injected line showed a change trend toward S-line. It has been reported that susceptibility to insecticides in *B*. *tabaci* is correlated with bacterial symbiont densities [33]. Thus, the fluctuation of bacterial abundance may pose a degree of impact on insecticide resistance in host insects. Additionally, *Gluconacetobacter* sp, which are belonged to acetic acid bacteria, have been shown to involve in the regulation of innate immune system homeostasis of insects [34]. This reveals a possible role of this bacterial genus in shifting host phenotype to a certain extent. However, *Gluconacetobacter*, as well as any other bacterial genera, were obviously less abundant than *Arsenophonus* in the injected homogenate from S-line insects (Fig 5C). And indeed, a significant change had been seen in the injected line with increasing proportion of S-type *Arsenophonus* strain (Fig 4B). From this perspective, the sensitized effect in injected line should be dominantly associated with S-type *Arsenophonus* rather than other co-infecting symbionts.

### Mechanisms underlying the sensitized effect conferred by S-type *Arsenophonus*

**Fig 6:**
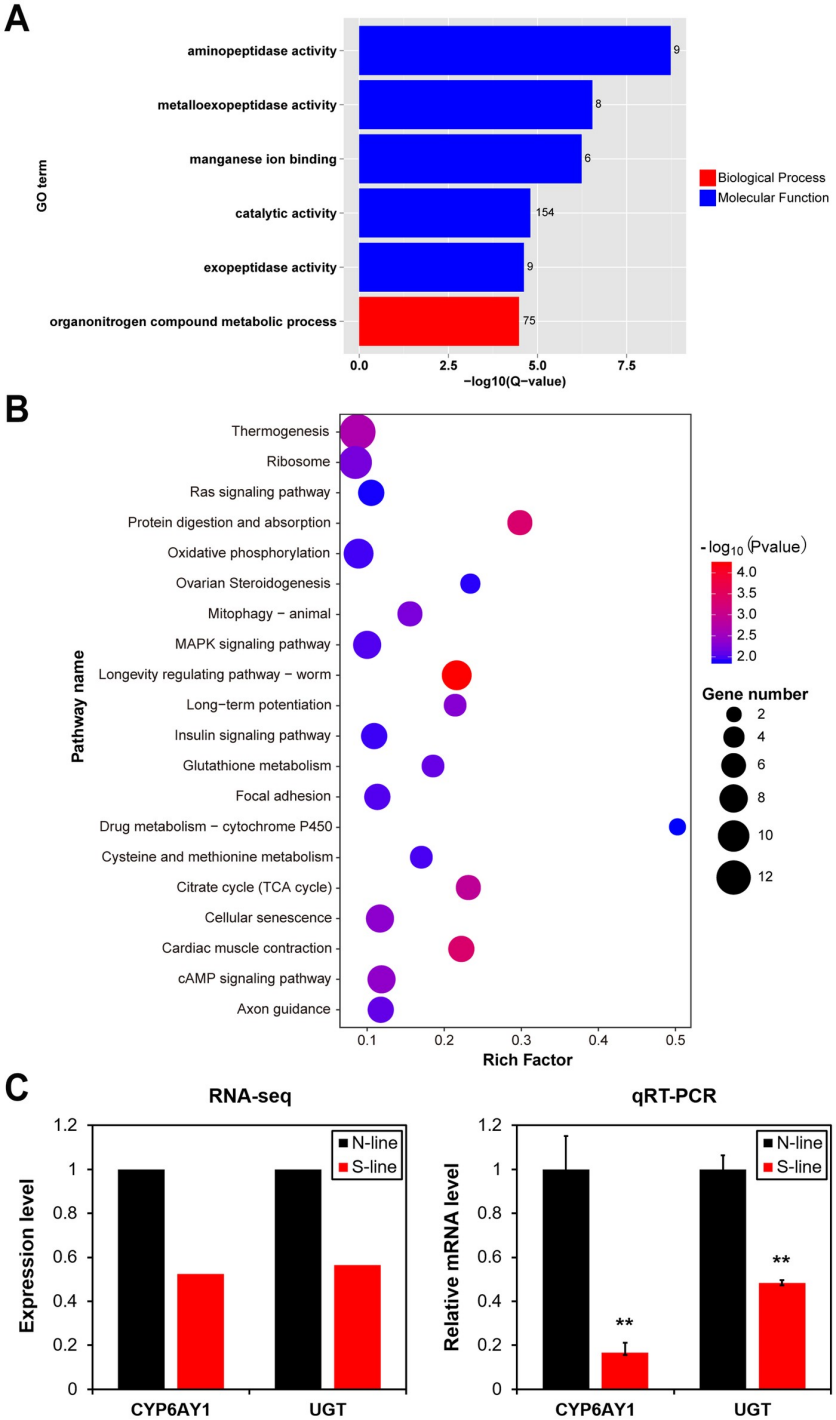
Comparison of the transcriptomes of *N*. *lugens* dominantly carrying S-type (S-line) and N-type (N-line) *Arsenophonus*. **(A)** GO enrichment analysis of differentially expressed genes (DEGs). Only the categories significantly enriched with down-regulated genes are presented. The number beside each column indicates the number of DEGs in the corresponding category. **(B)** The top 20 enriched pathways annotated in the KEGG database of DEGs. **(C)** The expression pattern of genes MSTRG.10290 (P450 CYP6AY1) and MSTRG.10418 (UDP-glucuronosyltransferase, UGT) revealed by RNA-seq analysis and validated by qRT-PCR. The qRT-PCR result is normalized relative to the *β*-actin mRNA level, and is shown by mean + SEM (n = 3). **: *P* < 0.01. (*t*-test).

To gain insight into the phenotypic host diversity conferred by different *Arsenophonus* strains, we compared gene expression patterns between the N-line and S-line *N*. *lugens*. A good consistency was observed from two duplications of the RNA-seq datasets from N-line and S-line *N*. *lugens* (S7 Fig), and the analysis revealed a total of 1,781 differentially expressed genes (DEGs), including 1,153 up- and 628 down-regulated genes in S-line group compared to N-line group (S8 Fig, S5 Table). Twenty randomly selected DEGs were validated by qRT-PCR, supporting the reliability of our RNA-seq analysis (S9 Fig). Grouping DEGs based on their GO annotations revealed that despite the higher proportion of up-regulated genes, preferential enrichment in any particular category was not observed and, in fact, they were evenly distributed across all functional categories. By contrast, down-regulated genes were enriched in the functional categories of catalytic activity, organonitrogen compound metabolic process, manganese ion binding, and several kinds of peptidase activity (adjusted *P*-value < 0.05, Benjamini and Hochberg method, Fig 6A). The suppressed functions associated with S-type *Arsenophonus* would be predicted to bring more specific changes to host insects than the enhanced functions. We also analyzed the pathways affected by the DEGs by annotating to the KEGG database, which showed that the majority of DEGs are involved in signal transduction pathways and metabolic pathways (Fig 6B, S6 Table). The affected pathways also included those related to environmental adaptation such as thermogenesis [35], insecticide metabolism by cytochrome P450 [36] and bioinsecticide resistance mediated by MAPK signaling pathway [37]. Thus, our functional analysis of DEGs is consistent with a potentially consequential effect of S-type *Arsenophonus* in *N*. *lugens* that could impact xenobiotics-induced response and catalysis.

Consistent with a negative effect on insecticide resistance in S-line insects, we found two genes MSTRG.10290 and MSTRG.10418, which were annotated as cytochrome P450 CYP6AY1 and UDP-glucuronosyltransferase (UGT), were significantly suppressed in S-line samples (Fig 6C). High expression of *CYP6AY1* plays a vital role in insecticide resistance of *N*. *lugens* and its protein product can efficiently metabolize insecticide [38,39]. Previous studies also showed that UGT plays an important role in the detoxification of xenobiotics and their toxic metabolites in insects [40,41]. Thus, the down-regulation of these genes in *N*. *lugens* carrying S-type *Arsenophonus* could be one of the major reasons for the decrease in insecticide resistance.

### Metabolic differences reveal as potential trade-off conferred by S-type *Arsenophonus*

During evolution, facultative symbionts and host species undergo a dynamic relationship [4,42]. On one hand, many symbionts evolve to broaden the diversity of host species. On the other hand, hosts may acquire multiple lineages of symbionts. These processes are enabled by vertical and horizontal transfer of symbionts within and among species [43]. Occasionally lineages confer a special phenotypic effect to the host, eg. the replacement of *Buchnera* with a special genotype increased the heat tolerance of the aphid [11]. Our study identified a specific host phenotypic change, namely sensitivity to chemical insecticide, due to the difference of the *Arsenophonus* strain. However, this change would confer an ecological disadvantage to host insects of S-type *Arsenophonus* in farming areas where pesticides are widely used. Because evolution of symbionts usually tends to enhance its transmission in host populations [44], we wondered whether there is any physiological or ecological compensation conferred by this type of bacterium in the absence of insecticide.

**Fig 7:**
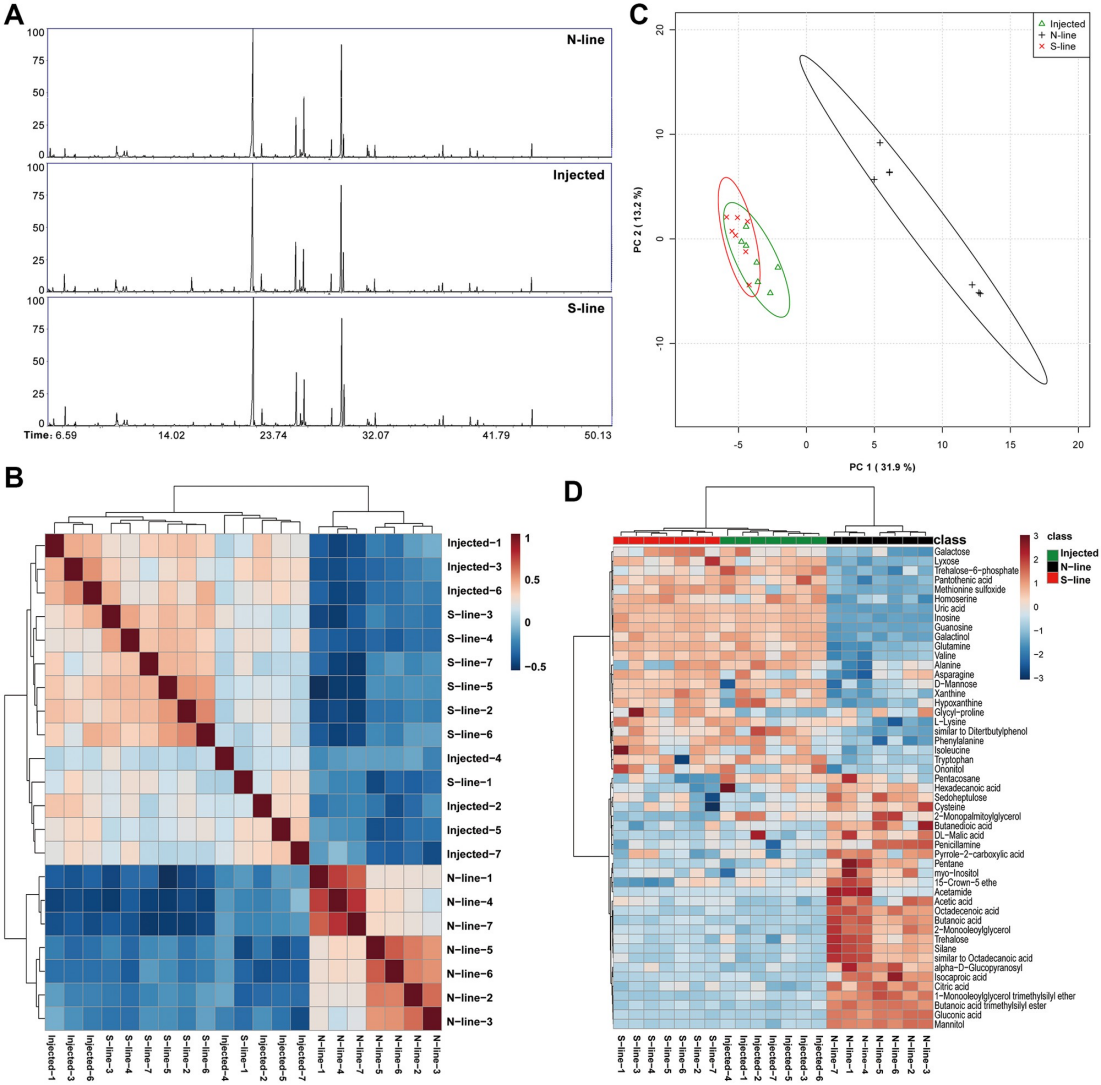
The impact of S-type *Arsenophonus* on the metabolic profile of *N*. *lugens*. **(A)** Representative total ion current chromatogram from N-line, S-line, and injected line samples. **(B)** The overall correlation heatmap of samples from three tested lines based on the variation in the metabolome. All samples are represented on both the x- and y-axis in the same order. The correlation was calculated according to Spearman’s rank correlation coefficient. **(C)** Principal component analysis (PCA) plots of metabolite composition of samples from three tested lines. Each symbol represents a sample, different symbol shapes denote different groups. The explained variances are shown in brackets. **(D)** Clustering of significant metabolites across three tested lines as determined by ANOVA analysis. The Euclidean distance measure and the ward.D clustering algorithm were used.

To explore this question, we compared the metabolome of insects from *N*. *lugens* S-line, N-line, and the injected line, by using gas chromatography-mass spectrometry (GC-MS). A total of 137 metabolites were identified across all samples (n = 7 per insect line) (Fig 7A). ANOVA analysis revealed 51 features that were different in abundance profiles across the samples (S7 Table). Eighty-two percent of the outlier metabolites were significantly different in comparisons of S-line to N-line and injected line to N-line, whereas only 12% of the outliers showed a significant difference between S-line and the injected line. Correlation analysis revealed that the injected line was more equivalent to S-line than to the N-line (Fig 7B). PCA analysis also supported the similarity in the metabolomes of the injected line and the S-line. Whereas there was a clear distinction between the N-line and the other two lines, no significant difference was observed between injected line and S-line (Fig 7C), reinforcing the success of *Arsenophonus* replacement by injection. Further analysis showed that carriers of the S-type *Arsenophonus* displayed 19 increased and 23 decreased metabolites (*p* < 0.05, Fig 7D, S7 Table). All differential metabolites between the S-line and N-line *N*. *lugens* were attributed to a total of 33 pathways (S10 Fig, S8 Table).

**Fig 8:**
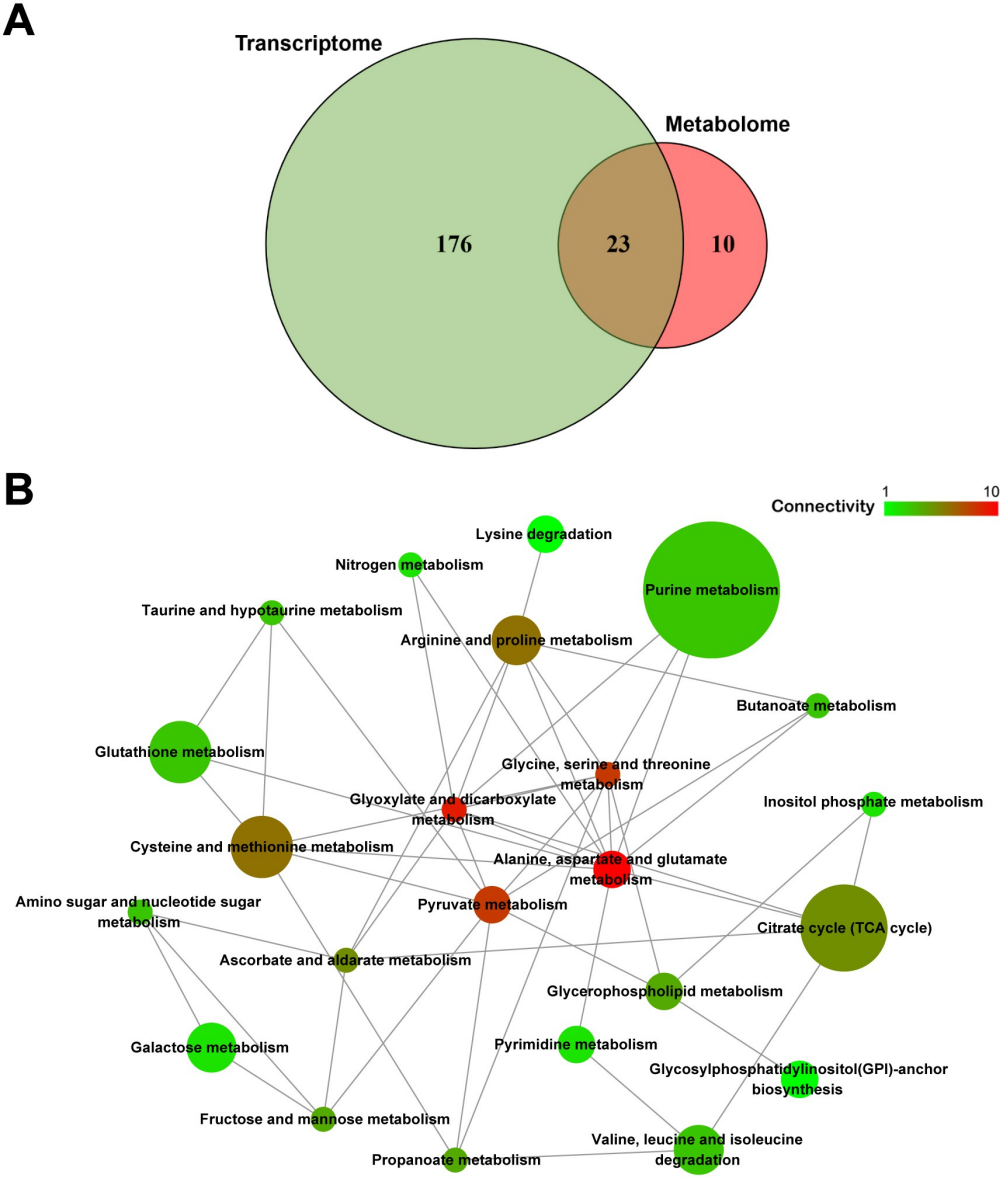
Integrated analysis of regulated pathways identified by transcriptome and metabolome analysis. **(A)** Venn diagrams of the regulated pathways identified from transcriptome and metabolome analysis. **(B)** Pathway-pathway network reflecting the diversity of the regulatory mode between N-type and S-type *Arsenophonus* and the connectivity to the host insect. The point size varies with the differential factors number in the corresponding pathway, and the point color varies with the connectivity of source pathway to its target pathways.

Further integrated analysis of transcriptomes and metabolomes revealed an overlap of 23 pathways (Fig 8A). The purine metabolism and citrate cycle (TCA cycle) pathways contained the most differential factors (S11 Fig). A pathway-pathway network based on the correlation between each pathway (Fig 8B) showed that the regulatory network differed between S-line and N-line insects. The patterns for DEGs and metabolites in these pathways suggest that S-type *Arsenophonus* is capable of decreasing carbohydrate metabolism in the *N*. *lugens* host (S12 Fig), which in turn leads to accumulation of amino acids including multiple essential amino acids such as methionine, valine, lysine, and phenylalanine (Fig 7D) [45]. Cases in which symbionts assist the host in the synthesis of essential amino acids are common [46,47]. Our results revealed that S-type *Arsenophonus* are more likely to provide amino acids to host insects than N-type *Arsenophonus*. We cannot definitively conclude that this represents a significant ecological compensation for reducing host resistance to chemical insecticide, or vice versa. One possibility is that the S-type *Arsenophonus* used to be a widespread strain that is well adapted to *N*. *lugens* and is quite far along the facultative to obligate evolutionary path. However, it has recently been replaced in most *N*. *lugens* populations by the N-type *Arsenophonus* strain that may be less good at providing nutritional benefits but improves survival under widespread insecticide selection. Nevertheless, the nutrient supplementary benefit should represent an important existential value in the evolutionary history of this symbiosis.

### Conclusion

In this study, we discovered a novel *Arsenophonus* strain that decreased insecticide resistance in the pest insect, *N*. *lugens*. Previous studies have mostly focused on the beneficial phenotypes that symbionts confer to their hosts [9,11,31]. Our findings validate a specific acquired phenotype mediated by symbionts, in that they may confer an ecological disadvantage in certain circumstances. We also attempted to isolate and *in vitro* culture both strains of *Arsenophonus* from *N*. *lugens* but failed just as was performed by Fan et al [22], and the short-fragment libraries for genomic resequencing made it hard to assemble a draft genome of S-type *Arsenophonus* for comparative genomic analysis in this study. In spite of that, we successfully reduced the insecticide resistance of the *N*. *lugens* line carrying resident N-type *Arsenophonus* by injecting homogenate containing S-type bacterium. This practice expands our thinking of developing methods to protect the world’s agriculture. Upon the improvement of infection approach, the usage of insecticide to control this pest insect will be dramatically reduced, which will certainly benefit the agroecosystem. In other way, the symbiotic relationship between *Arsenophonus* and *N*. *lugens* is of significant scientific interest. In-depth insight into the history of infections with different *Arsenophonus* strain in this insect will help to understand the co-evolution of symbionts and eukaryotes in an ecological environment affected by human activities.

## Materials and methods

### Insect sampling and bioassays

Individuals from various populations of *N*. *lugens* were collected from 2009 to 2015 in different locations of Asia (S2 Table, Fig 2). Some populations were stored in 95% ethanol, while some were kept alive and reared on the Fenghuazhan rice variety (a sensitive variety).

Screening for insecticide-resistant and -susceptible individuals was performed on a field-collected population from Guangxi, China in 2011. We used the rice stem dipping method to test the insecticide resistance level of this population as described [39]. The insecticide imidacloprid and buprofezin were dissolved in acetone and diluted to a series of concentrations, followed by adding 1% Tween 80. Rice stems rooted in culture cups were then dipped into the insecticide solutions for 30 s. After the rice stems had been air dried, 20 third instar nymphs were placed into each culture cup. Each treatment was performed in triplicate. After 96 h, nymph mortality was calculated. After the LC-P line was obtained, third instar nymphs of the second generation were treated with LC_90_ and LC_10_ concentrations of each insecticide. Those individuals that survived LC_90_ treatment were considered resistant, whereas those that died under LC_10_ treatment were considered susceptible. The untreated individuals were used as CK control.

### Genome resequencing and sweep mapping analysis

Sixty individuals from each treatment were separated randomly into two duplicates. The genomic DNA of each library was extracted as previously described [39]. Each DNA sample was fragmented to 500 bp by a Covaris sonicator and used to generate Illumina libraries. Whole genomes were sequenced on the Illumina Hiseq2000 platform in BGI-Shenzhen (Shenzhen, China), which produced 100-bp paired-end reads. The clean sequencing reads were aligned to the reference genomes of *N*. *lugens* (GCF_000757685.1) and *Arsenophonus nilaparvatae* (GCF_000757905.1) using the Burrows-Wheeler Alignment Tool (BWA, V.0.7.13) [48]. The Picard software package (http://broadinstitute.github.io/picard/) was used to identify duplicate reads and create BAM indexes. The best practices guide recommended by the Genome Analysis Toolkit (GATK) was followed to call and refine the SNP variants [49,50]. The SNPs with high quality were annotated by SnpEff software [51]. The Blast2GO software was used to obtain Gene Ontology (GO) annotation of the whole coding genes and PTC genes in the *Arsenophonus* genome [52], and enrichment analysis was performed based on the Fisher’s exact test with Benjamini and Hochberg (BH) adjustment. Finally, VCFTools software (V.0.1.15) was used to estimate the pairwise *F*_ST_ values and genome-wide linkage disequilibrium (LD) values [53], and Samtools software (V.1.7.1) was used to calculate the genome-wide distribution of sequencing depth [54].

### *Arsenophonus* genotyping

Genomic DNA from *N*. *lugens* individuals was used as the template for PCR amplification. Primers were designed from five *Arsenophonus* genomic fragments ranging from 250 bp to 600 bp, and each contained multiple SNPs. Details of genomic fragments are shown in S9 Table, and all primers are listed in S10 Table. After amplification with KAPA2G Fast Genotyping mix according to the user guide (Massachusetts, USA), the PCR products were sent for Sanger sequencing by IGE Biotechnology Ltd (Guangzhou, China). The sequencing chromatograph was analyzed using Chromas software (Technelysium Pty Ltd, South Brisbane, Australia).

### Replacement of resident *Arsenophonus*

We used an improved method of homogenate injection to replace the resident *Arsenophonus* in the N-line with S-type bacterium according to the approach described by Moran and Yun [11]. The injection experiment was conducted with 3.5 Drummond needles and a microinjector (NARISHIGE I M-31, Nikon, Japan). This injection protocol has been proved to have no negative effect on host’s phenotypes in several studies [39,40,55]. Briefly, thirty to ninety newly emerged females from the N-line were injected with the homogenate from the S-line, and each injected female was introduced into a separate plastic cage with fresh rice for oviposition. The *Arsenophonus* genotyping was conducted 7 days after injection. All the offspring of injected females containing S-type bacterium were pooled together and considered as F1. When the F1 insects grew to female adults, we repeated the process of injection and genotyping. Again, the progeny of injected females containing S-type bacterium was pooled as F2. Following this procedure, the injection and genotyping were repeated 8 times in total to generate the injected insect line of F8. The insecticide resistance levels were tested in third instar nymphs of F2 and F8 according to the bioassay method described above.

#### Bacterial community analysis

Every five 1-day-old adult females from each insect line at F8 were pooled as one sample, and surface-sterilized with 70% ethanol for 1 min, 10% sodium hypochlorite for 1 min and three washes of ultrapure water for 1 min. Six duplicate samples were prepared for each line. DNA was extracted from washed sample using the MinkaGene Stool DNA Kit (mCHIP BioTech CO., LTD, Guangzhou, China) according to the manufacturer’s instruction. PCR amplicon libraries were constructed using bacterial primers 341F (5′-CCTAYGGGRBGCASCAG-3′) and 806R (5′-GGACTACHVGGGTWTCTAAT-3′) targeting the V3 + V4 hypervariable regions of the 16S rRNA genes. PCR reactions, containing 25 μl 2x Premix Taq (Takara Biotechnology, Dalian Co. Ltd., China), 1 μl each primer(10 mM) and 3 μl DNA (20 ng/μl) template in a volume of 50 μl, were amplified by thermocycling: 5 min at 94°C for initialization; 30 cycles of 30 s denaturation at 94°C, 30 s annealing at 52°C, and 30 s extension at 72°C; followed by 10 min final elongation at 72°C.

DNA libraries were generated from purified PCR products using NEBNext Ultra DNA Library Prep Kit for Illumina (New England Biolabs, MA, USA). High-throughput sequencing was performed by the Guangdong Magigene Biotechnology Co., Ltd. (Guangzhou, China) on the Illumina Hiseq2500 platform with 250-bp paired-end mode. Clean reads were merged using FLASH (V1.2.11) with allowable error ratio of the overlap region lower than 0.1 [56], and sequences analysis were performed by Usearch software (V8.0.1517) at an identity threshold of 97% similarity [57]. For each representative sequence, the Silva (https://www.arb-silva.de/) database was used to annotate taxonomic information (the confidence threshold ≥ 0.5). Alpha (Chao1, Shannon, Simpson, and dominance) and β diversity (PCoA and Anosim) were performed with QIIME (Version 1.9.1) and displayed with R software (Version 2.15.3) [58]. Significant differences in the relative abundance of bacterial genera across different insect lines were analyzed by using one-way analysis of variance (ANOVA) and Tukey’s range test.

### RNA-seq and differential expression analysis

Total RNA was extracted from 1-day-old adult females of the S-line and N-line *N*. *lugens* individuals using total RNA extract kit (Omega, Norcross, GA, USA). The cDNA libraries from two duplicates of each line were constructed and sequenced by Vazyme Biotech Co.,Ltd (Nanjing, China) on the Illumina Hiseq Xten platform. All clean reads were aligned to the *N*. *lugens* genome by Hisat2 software (v.2.1.0) [59]. Assembly of the aligned reads was performed using StringTie software (v.1.3.4) according to the reference gff file [60]. To give insight into the changes in insect host as much as possible, the differentially expressed genes between the S-line and N-line samples were estimated by DESeq software in R based on a relatively loose threshold of log_2_ ratio ≥ 0.8 and adjusted *P*-value ≤ 0.05 (BH adjustment). The whole transcripts were annotated by BlastX in the National Center for Biotechnology Information (NCBI) database. The GO enrichment was performed as above, and the GO annotation of whole transcripts of *N*. *lugens* was used as the background. The pathways of transcripts were annotated in the Kyoto Encyclopedia of Genes and Genomes (KEGG) database by KEGG Automatic Annotation Server (KAAS).

To support the transcriptome analysis, twenty DEGs were randomly selected for measurement of expression levels by quantitative real-time PCR (qRT-PCR) in LightCycler480 (Roche, Indianapolis, IN, USA) using GoTaq qPCR Master Mix (Promega, Madison, WI, USA). The primers used for qRT-PCR are listed in S10 Table. The amplification conditions were: 95 °C for 10 min, followed by 45 cycles of 95 °C for 10 s, 60 °C for 20 s, and 72 °C for 20 s. The *β*-actin gene was used as an internal control [55]. For analysis, three independent biological samples from the S-line and N-line were tested.

### Metabolome assay

For metabolite extraction, 6 newly emerged females from each insect line were pooled together and ground into powder with an electronic grinding machine. The precooled methanol/water (2:1) solvent was added and the mixture was vortexed for 30s. After ultrasonic treatment in an ice bath for 15 min, the homogenized samples were centrifuged at 20 000 ×g for 10 min at 4°C. The supernatants from the same sample were mixed uniformly followed by adding 60 μL aqueous solution of ribitol (internal standard, 0.2 mg/mL). The resulting extraction was concentrated and dried under nitrogen before derivatization. For derivatization, the dried extraction was re-dissolved by adding 70 μL methoxy-amino-hydrochloride in pyridine (20 mg/mL). After vortexing, the mixture was incubated on a shaking table (200r/min) for 1.5h at 37 °C. Then, 100 μL N-Methyl-N-(trimethylsilyl) trifluoroacetamide (MSTFA) was added, and the reaction system was maintained at 37 °C for 30min. After centrifugation at 13,000 × g for 3 min, the supernatant was transferred to a linear tube in preparation for GC-MS analysis. Seven biological replications were performed for each insect line. In addition, three blank controls with only derivatization reagent were handled with the same method.

Compounds in the test and blank samples were separated and characterized with a DB-5ms UI column (length 30 m, I.D. 25 mm, Agilent) on an Agilent 7890A GC equipped with an Agilent 5975C VL MSD detector (Agilent Technologies). First, the sample solution was infused to the injector in splitless mode at 280 °C. Then the temperature programming was performed as follows: initial 70 °C temperature of the oven was held for 5 min followed by a rapid increase to 300 °C at the rate of 5 °C per minute, then held for 5min. Helium was used as a gas carrier, and its flow rate through the column was 1mL/min. In the range of 50–450 m/z, full scan mass spectra were ultimately obtained at the electron impact ionization (EI) of 70 eV. Spectral deconvolution and calibration were performed with AMDIS. To avoid false positives, the peaks with a signal-to-noise ratio (S/N) lower than 30 were excluded [61]. Retention time and compound peaks were then normalized to the ribitol internal standard. Artifact peaks were removed according to the blank controls. The identification of metabolites was made through retrieving their mass spectra in the NIST 8.0 (National Institute of Standards and Technology, USA) library with a threshold of match value ≥ 750, reverse match value ≥ 800, and a probability ≥ 60% [62]. Based on the quality and peak area of the added internal standard of ribitol, a matrix on the information of metabolites abundance for each sample was generated.

### Bioinformatics analysis

The difference in metabolite contents between two groups was calculated using ANOVA and the Mann–Whitney U-test in SPSS 13.0. Results were considered statistically significant if the *P* value was lower than 0.05. The online website of MetaboAnalyst (www.metaboanalyst.ca/) was further used to analyze the hierarchical cluster analysis (HCA) within all samples and overlapped metabolites. Principal component analysis (PCA) was conducted to investigate the relationship of test samples. For the pathway enrichment analysis, the differential metabolites between tested groups were assigned to metabolic pathways using a tool of MetPA in MetaboAnalyst. The value of -log(*P*) and impact value of each metabolic pathway was calculated using Hypergeometric Test. All the KEGG xml files (KGML) for the overlapped pathways from transcriptome and metabolome analysis were downloaded, and the interactions of these pathways were estimated with a custom Perl script according to the method described by Chen et al [63]. Finally, the pathway-pathway network was visualized using Cytoscape software (V.3.6.0).

## Supporting information

S1 Fig The distribution of average *F*_ST_ values across *Arsenophonus* genomes from resistant and susceptible *N*. *lugens* in 5-kb windows with 1-kb sliding steps. (TIF)

S2 Fig Alignment of sequences of *Arsenophonus* amplified from insecticide-resistant (IR) and insecticide-susceptible (IS) *N*. *lugens* to the reference sequences. (TIF)

S3 Fig Divergence of *Arsenophonus* genomes. (A) Pairwise genetic differentiation (*F*_ST_) between *Arsenophonus* genomes from buprofezin-resistant and -susceptible *N*. *lugens* in 5-kb windows with 1-kb sliding steps. (B) Pairwise genetic differentiation (*F*_ST_) across *Arsenophonus* genomes from buprofezin and imidacloprid-resistant samples (orange), and buprofezin and imidacloprid-susceptible samples (cyan) in 5-kb windows with 1-kb sliding steps. BR: Buprofezin-resistant, n = 2; BS: Buprofezin-susceptible, n = 2; IR: imidacloprid resistant, n = 2; IS: imidacloprid susceptible, n = 2.
(TIF)

S4 Fig GO enrichment of the genes containing a premature termination codon (PTC) in the *Arsenophonus* genome from susceptible *N*. *lugens*. (TIF)

S5 Fig Frequency of reference alleles in *N*. *lugens* considered to infect S-type *Arsenophonus* host insects. (TIF)

S6 Fig OTU abundances and taxonomic classifications between each insect line at different levels. (A) Phylum level. (B) Class level. (C) Order level. (D) Family level. (E) Genus level.
(TIF)

S7 Fig The correlation heatmap of the RNA-seq datasets from N-line and S-line *N*. *lugens*. The correlation was calculated according to Spearman’s rank correlation coefficient. S:S-line; N: N-line.
(TIF)

S8 Fig Volcano plots revealing genes that differ significantly between the S-line and N-line *N*. *lugens*. Dashed lines indicate the threshold value of significance.
(TIF)

S9 Fig Heatmap correlating comparative results from qRT-PCR and RNA-seq analyses. Positive and negative values in the heatmap mean up or down-regulated gene expression levels in the S-line compared to the N-line.
(TIF)

S10 Fig KEGG pathway annotation of differential metabolites between N-line and S-line *N*. *lugens*. The point size represents the significant compound number in the corresponding pathway.
(TIF)

S11 Fig Comparison of the number of affected components in the overlapping pathways identified by transcriptome and metabolome analyses. (TIF)

S12 Fig Carbohydrate metabolism-related pathways affected by S-type *Arsenophonus* infection. Pathways include pyruvate metabolism (A) and citrate cycle (TCA cycle) (B). Red indicates that the gene is up-regulated, blue indicates that the gene is down-regulated, while green indicates that the factor contains both up- and down-regulated genes.
(TIF)

S1 Table Genome-wide statistics of resequencing data of buprofezin-resistant and -susceptible samples. (XLSX)

S2 Table The *Nilaparvata lugens* populations used in this study. (XLSX)

S3 Table The proportion of infected *Arsenophonus* types in the Philippines population. (XLSX)

S4 Table Statistics for the relative abundance of core bacterial genera in each sample. (XLSX)

S5 Table Information of all differentially expressed genes. (XLSX)

S6 Table KEGG pathway annotation of differential expressed genes (DEGs). (XLSX)

S7 Table Compounds that differ in abundance profiles across different groups by ANOVA analysis. (XLSX)

S8 Table Pathway analysis of differential metabolites. (XLSX)

S9 Table Information of genomic regions for *Arsenophonus* genotyping. (XLSX)

S10 Table The primers used in this study. (XLSX)
