# Supplementary figures and images for: A distinct strain of Arsenophonus symbiont decreases insecticide resistance in its insect host
Source: PLoS Genet. 2018 Oct 17;14(10):e1007725. doi: 10.1371/journal.pgen.1007725 (PMC6205657; doi:10.1371/journal.pgen.1007725)

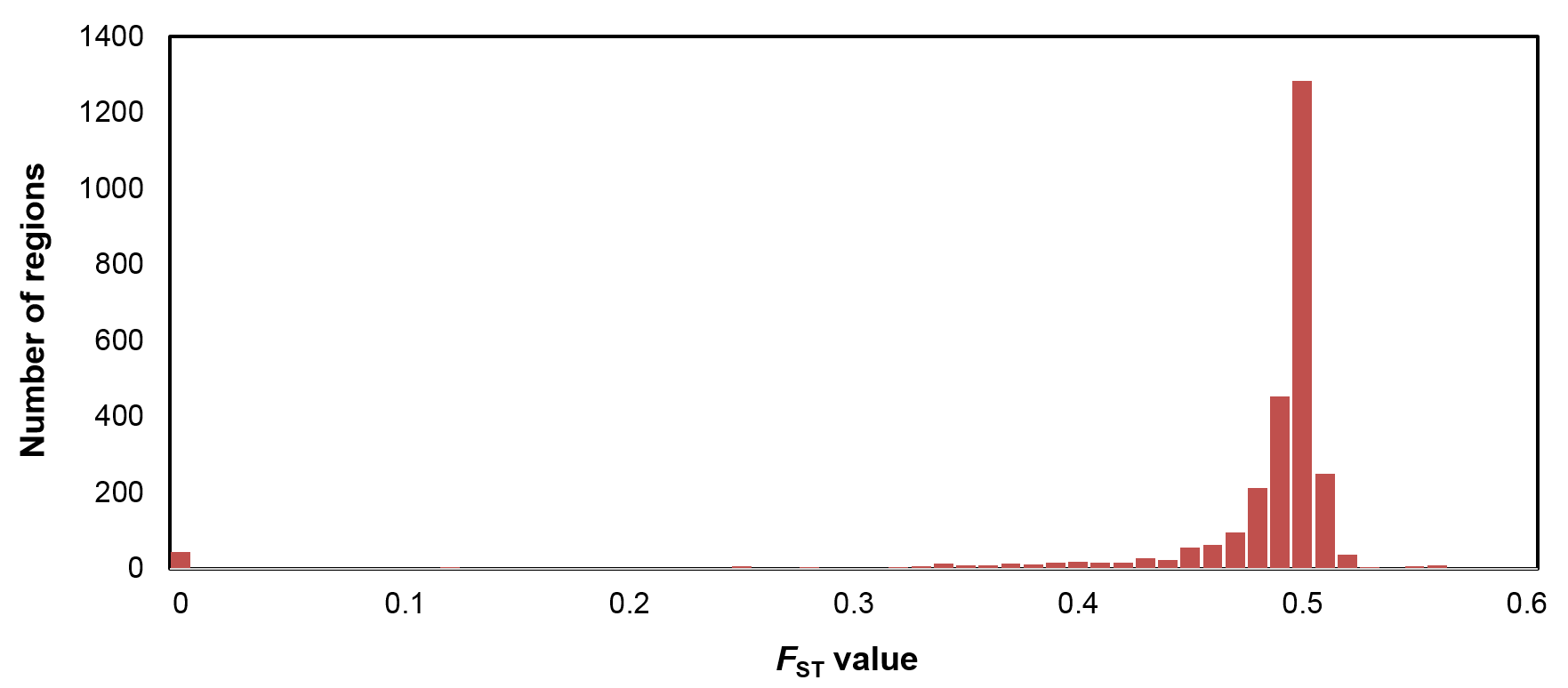

Supplement: S1 Fig — (TIF) [file pgen.1007725.s001.tif]

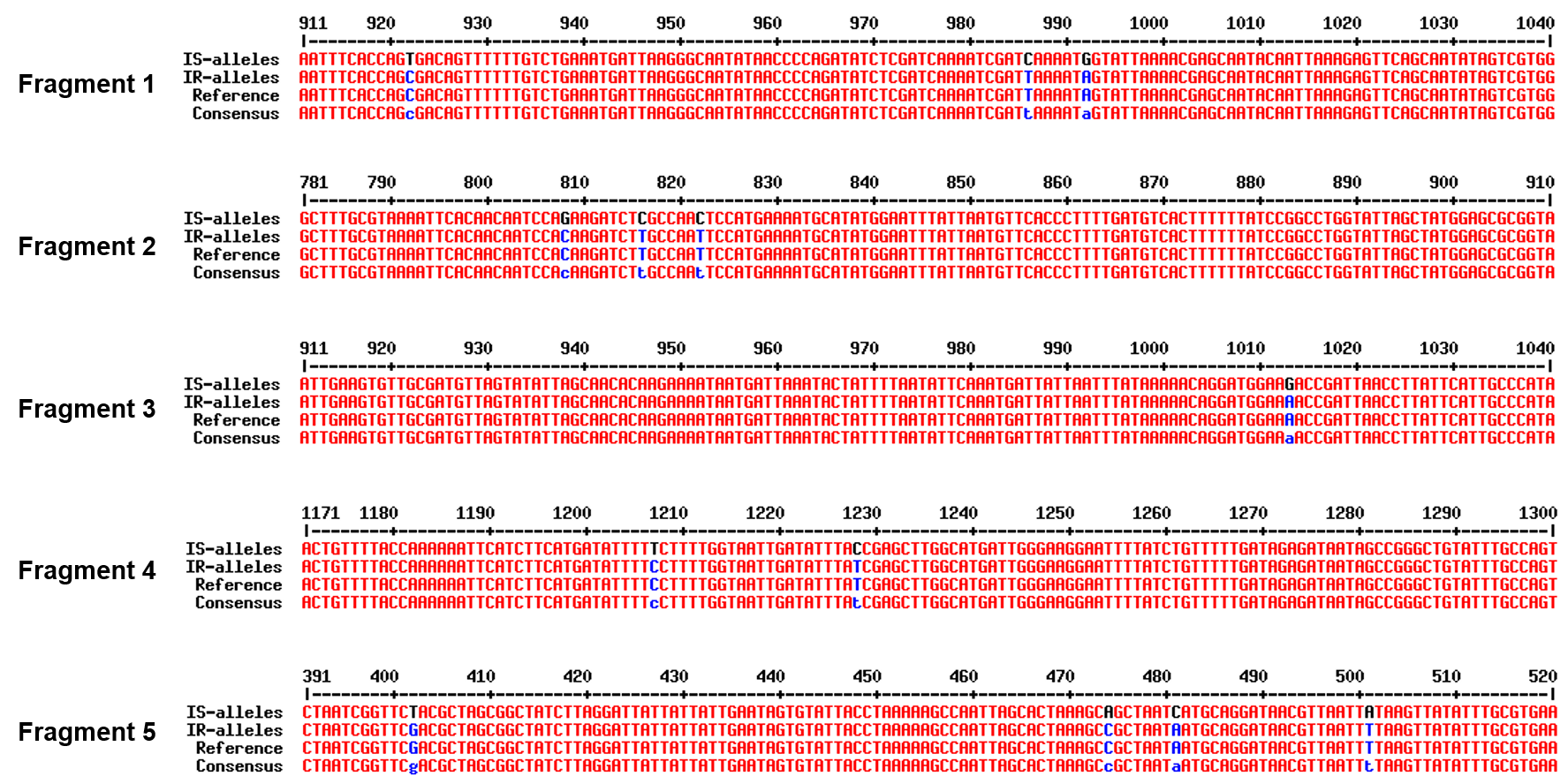

Supplement: S2 Fig — (TIF) [file pgen.1007725.s002.tif]

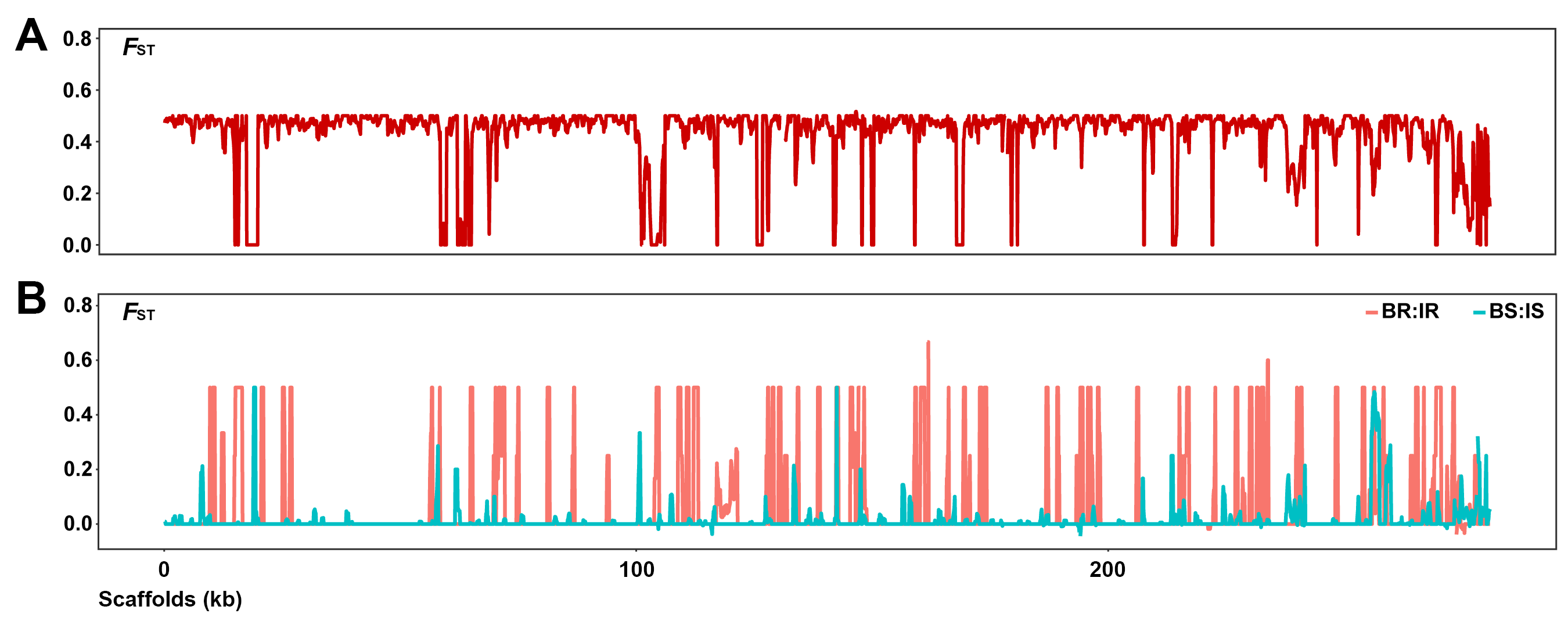

Supplement: S3 Fig — (A) Pairwise genetic differentiation (FST) between Arsenophonus genomes from buprofezin-resistant and -susceptible N. lugens in 5-kb windows with 1-kb sliding steps. (B) Pairwise genetic differentiation (FST) across Arsenophonus genomes from buprofezin and imidacloprid-resistant samples (orange), and buprofezin and imidacloprid-susceptible samples (cyan) in 5-kb windows with 1-kb sliding steps. BR: Buprofezin-resistant, n = 2; BS: Buprofezin-susceptible, n = 2; IR: imidacloprid resistant, n = 2; IS: imidacloprid susceptible, n = 2. (TIF) [file pgen.1007725.s003.tif]

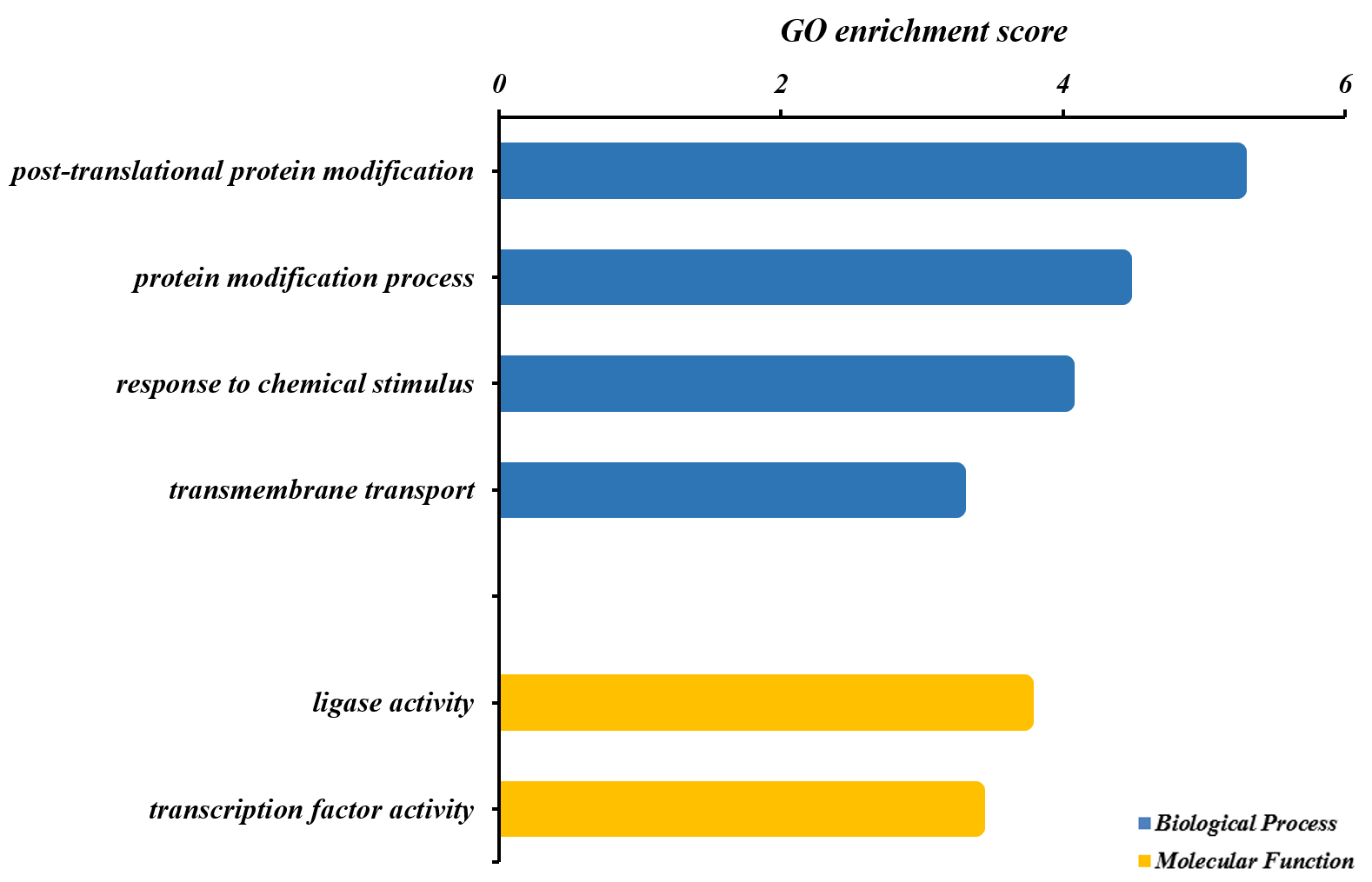

Supplement: S4 Fig — (TIF) [file pgen.1007725.s004.tif]

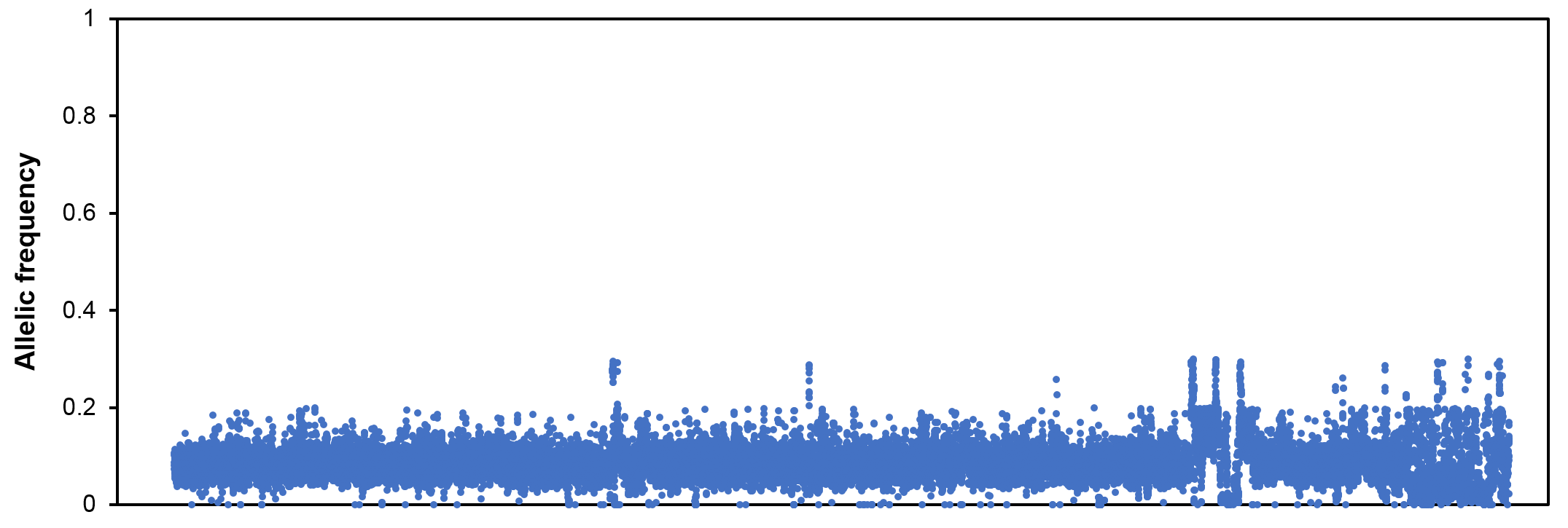

Supplement: S5 Fig — (TIF) [file pgen.1007725.s005.tif]

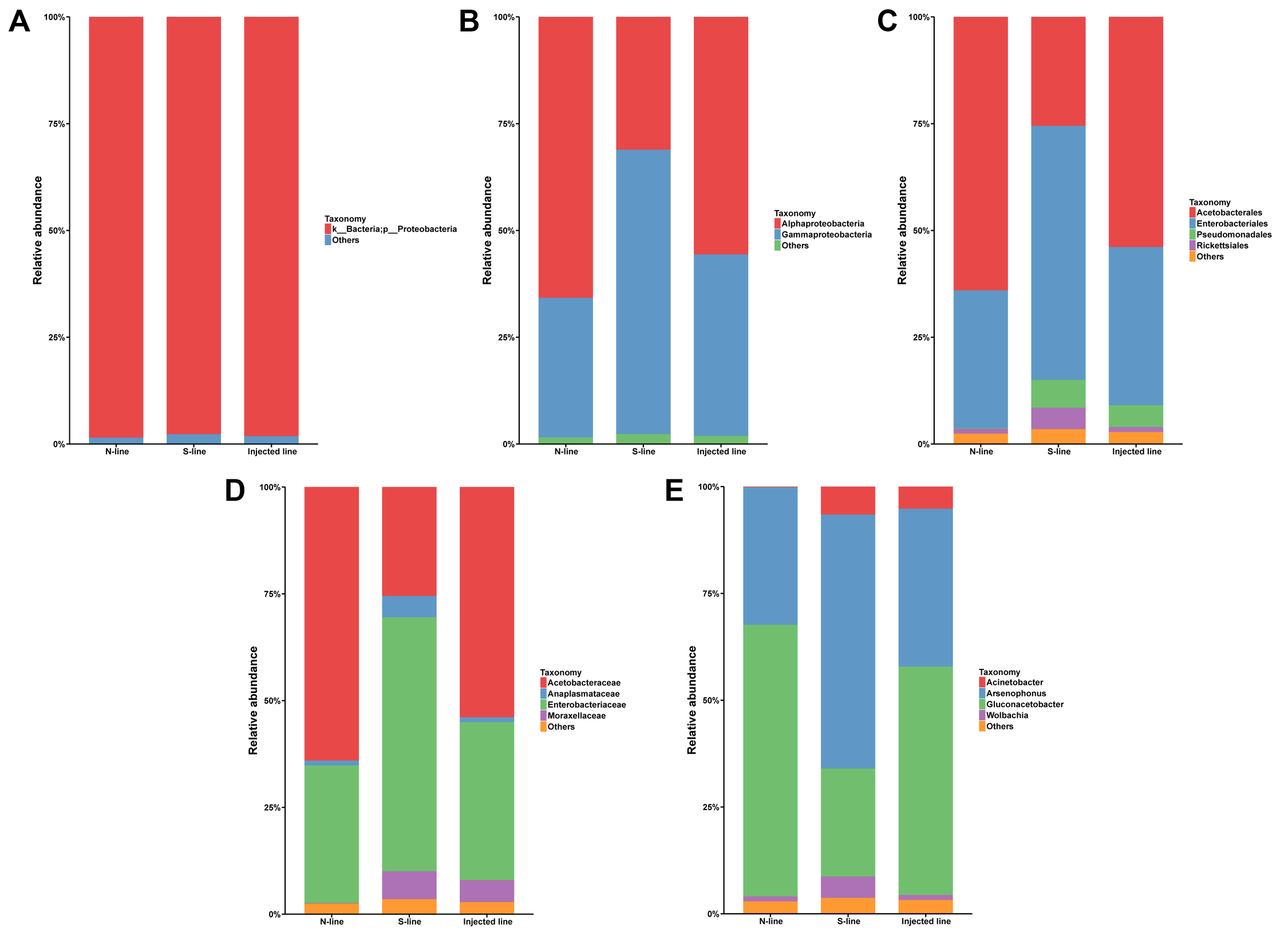

Supplement: S6 Fig — (A) Phylum level. (B) Class level. (C) Order level. (D) Family level. (E) Genus level. (TIF) [file pgen.1007725.s006.tif]

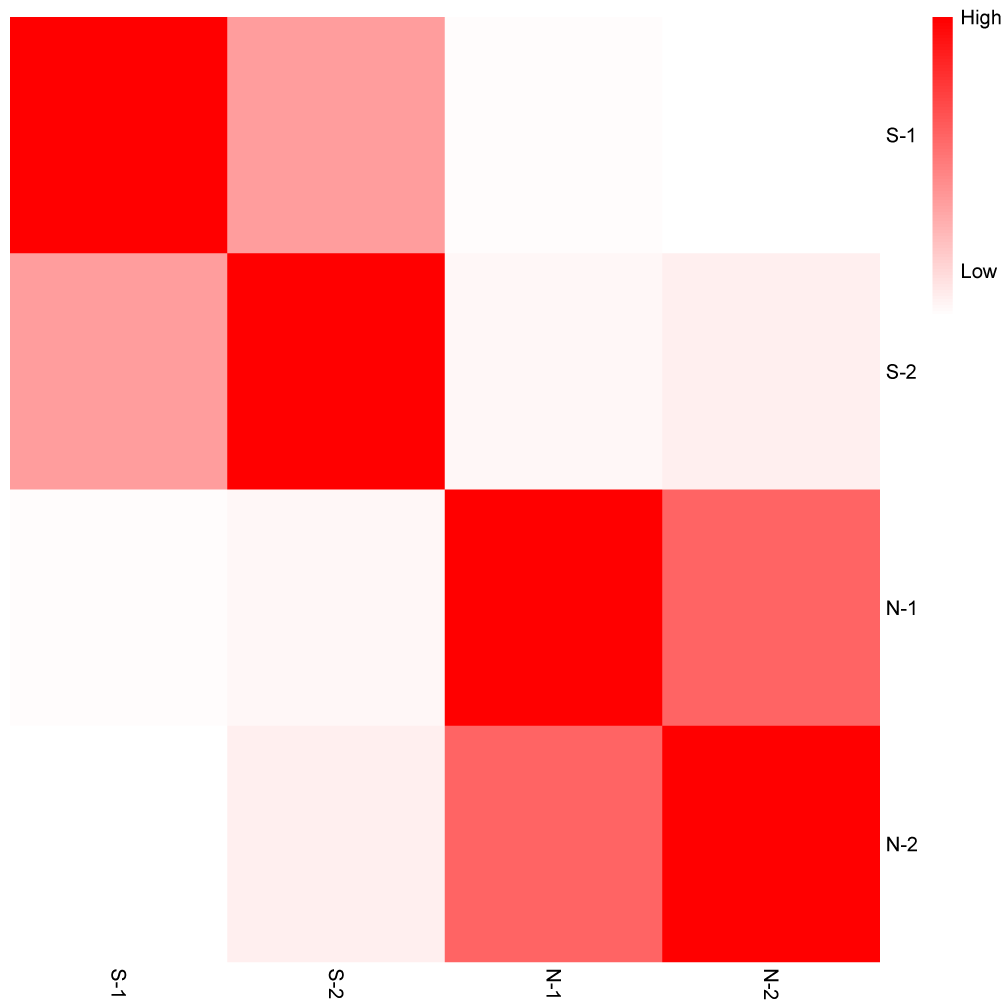

Supplement: S7 Fig — The correlation was calculated according to Spearman’s rank correlation coefficient. S:S-line; N: N-line. (TIF) [file pgen.1007725.s007.tif]

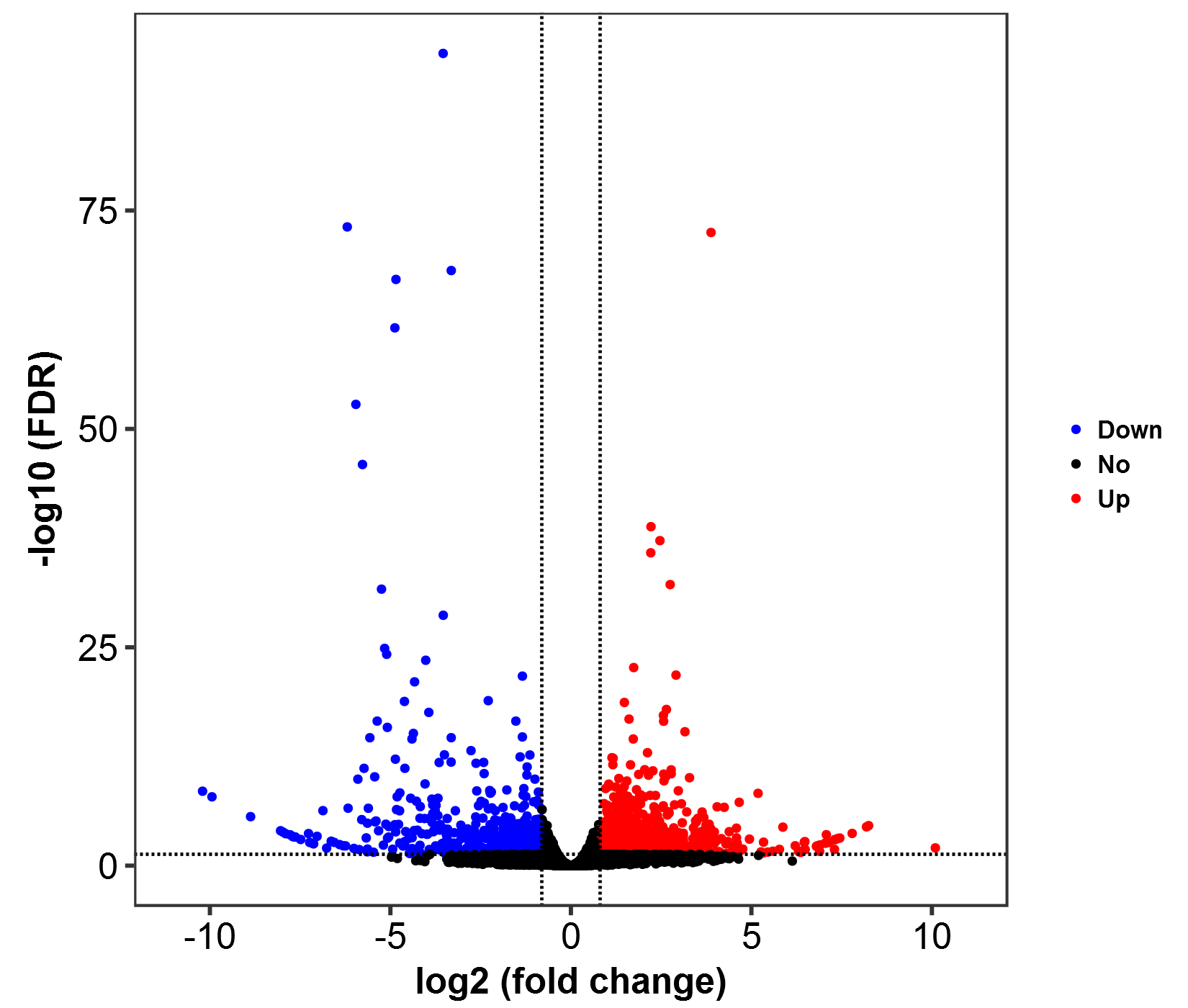

Supplement: S8 Fig — Dashed lines indicate the threshold value of significance. (TIF) [file pgen.1007725.s008.tif]

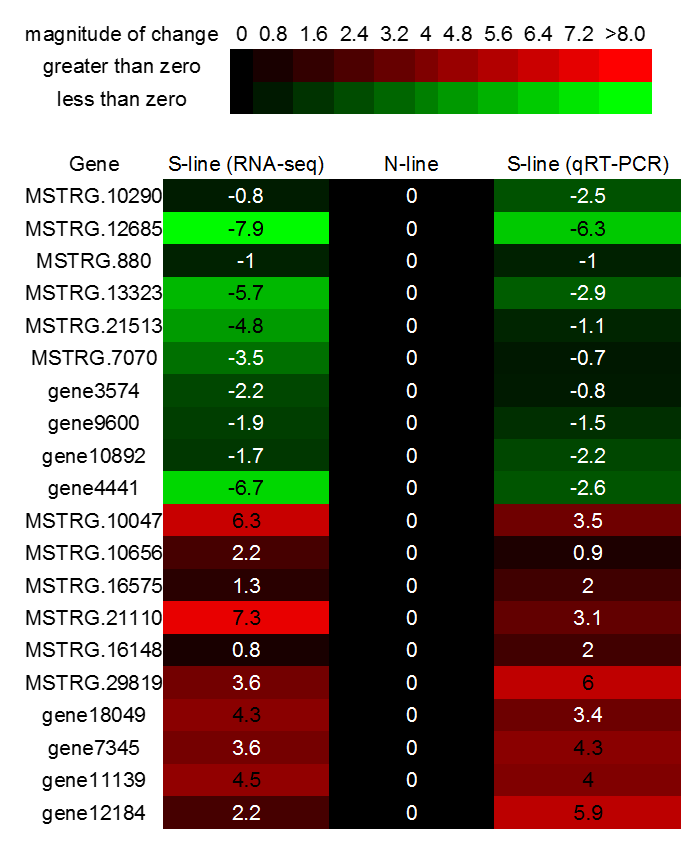

Supplement: S9 Fig — Positive and negative values in the heatmap mean up or down-regulated gene expression levels in the S-line compared to the N-line. (TIF) [file pgen.1007725.s009.tif]

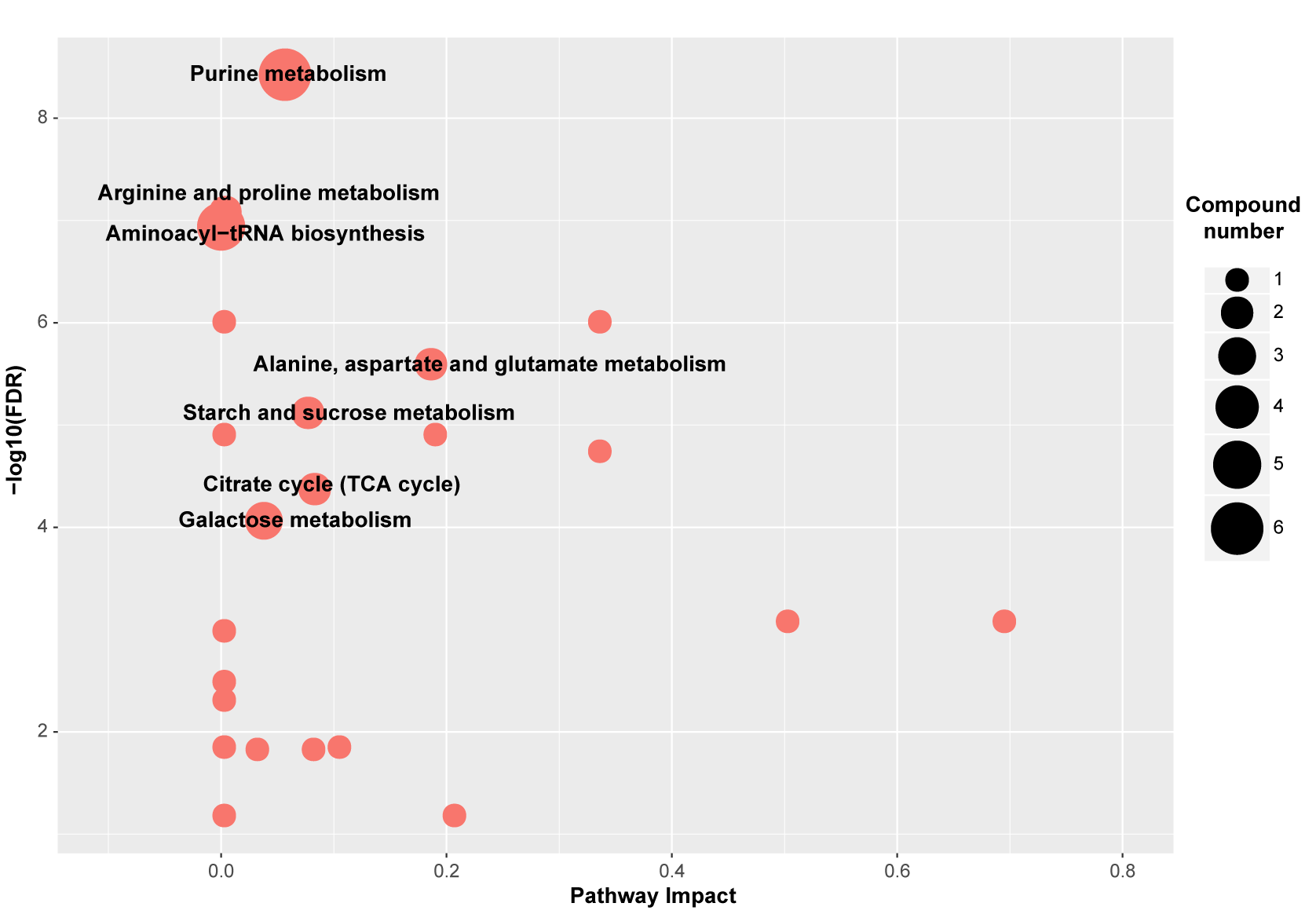

Supplement: S10 Fig — The point size represents the significant compound number in the corresponding pathway. (TIF) [file pgen.1007725.s010.tif]

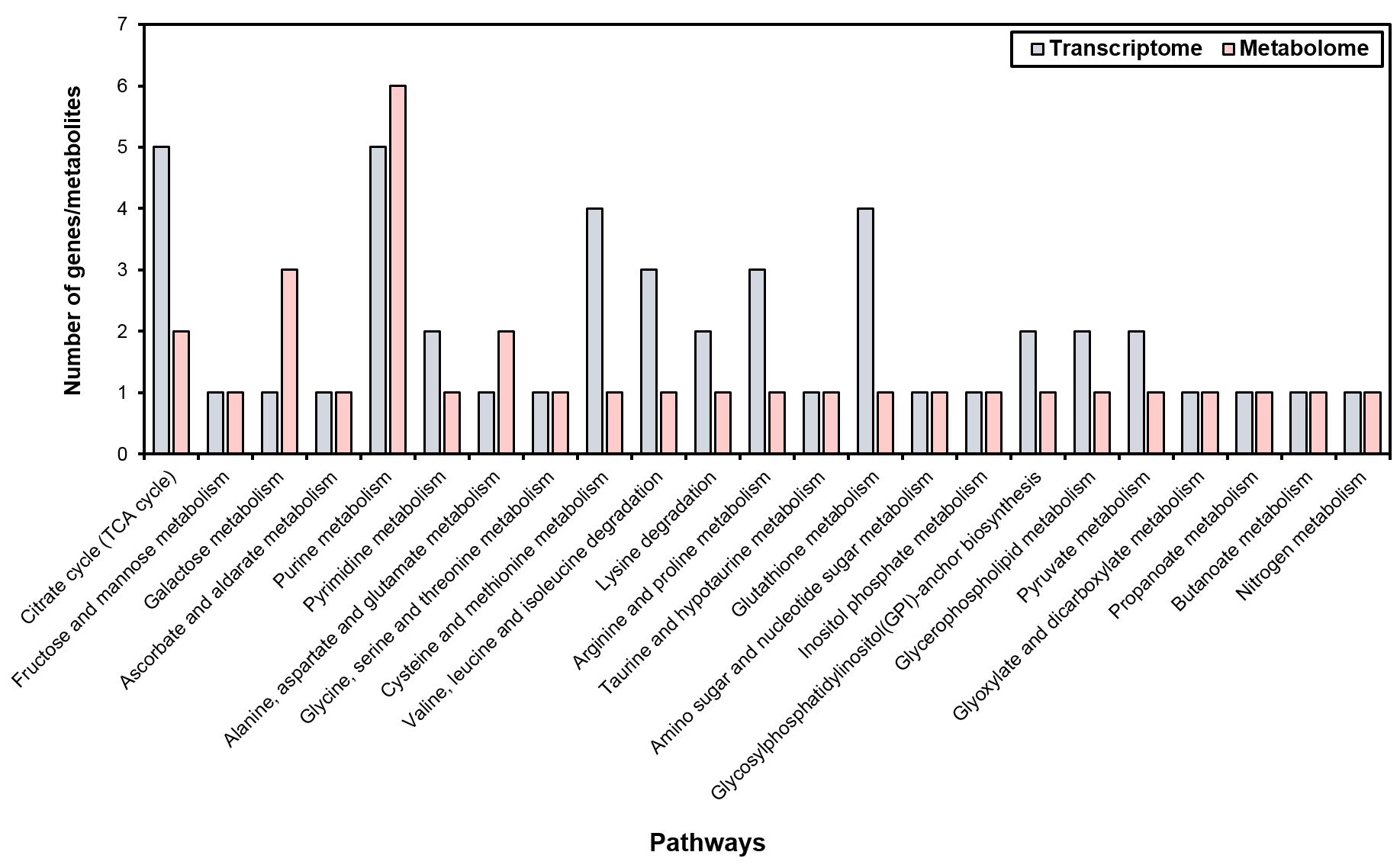

Supplement: S11 Fig — (TIF) [file pgen.1007725.s011.tif]

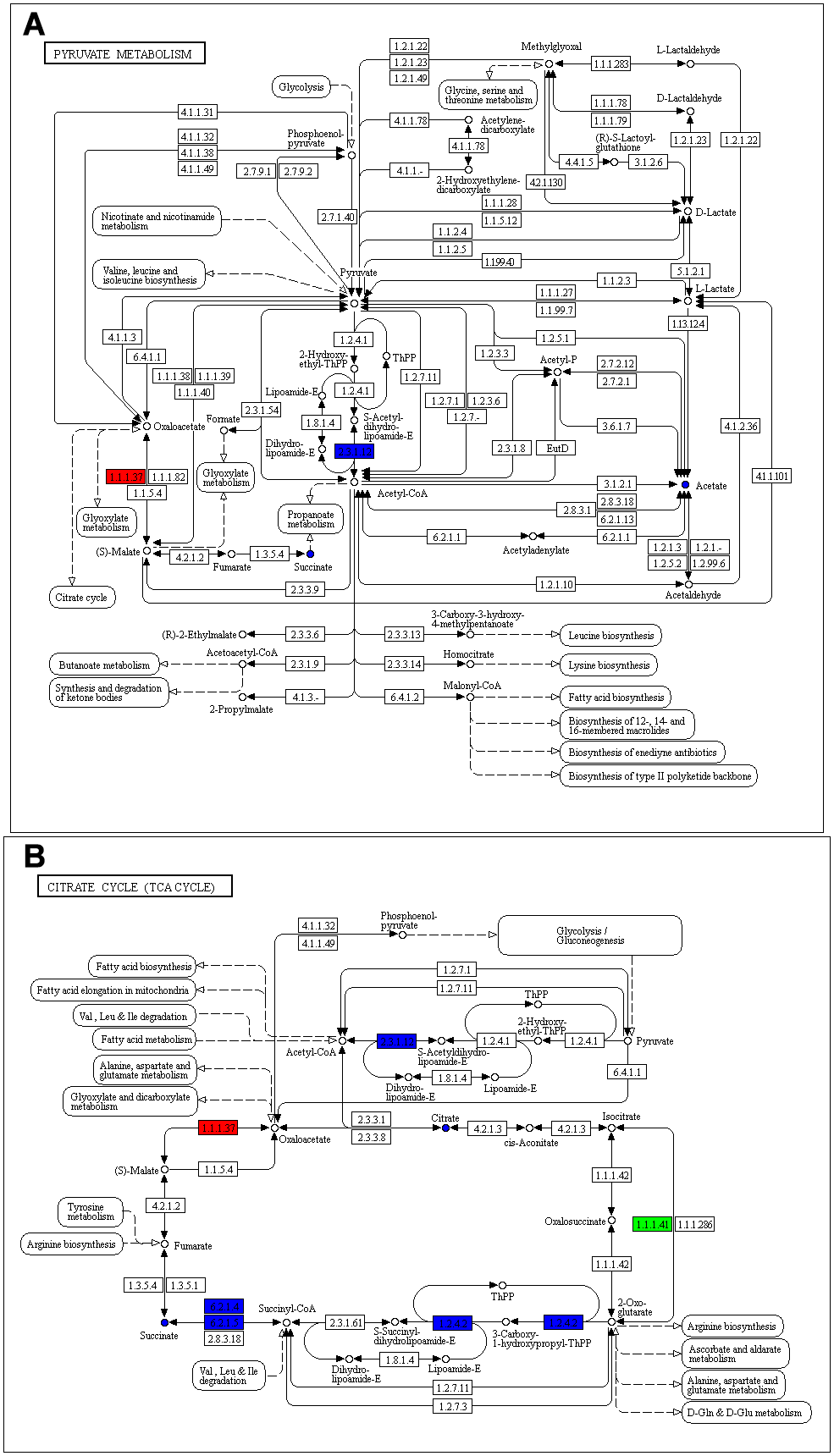

Supplement: S12 Fig — Pathways include pyruvate metabolism (A) and citrate cycle (TCA cycle) (B). Red indicates that the gene is up-regulated, blue indicates that the gene is down-regulated, while green indicates that the factor contains both up- and down-regulated genes. (TIF) [file pgen.1007725.s012.tif]
